# Genetic and Epidemiological Insights Into Respiratory Syncytial Virus Infections: A Comparative Study of Hospitalized Versus Community Cases in Portugal (2021–2023)

**DOI:** 10.1111/irv.70147

**Published:** 2025-10-13

**Authors:** Miguel Lança, Vânia Gaio, Ana Paula Rodrigues, Camila Henriques, Licínia Gomes, Daniela Dias, Maria de Jesus Chasqueira, Raquel Guiomar, Aryse Melo

**Affiliations:** ^1^ National Reference Laboratory for Influenza Virus and Other Respiratory Virus National Institute of Health Doutor Ricardo Jorge Lisboa Portugal; ^2^ NOVA Medical School, Faculdade de Ciências Médicas Universidade NOVA de Lisboa Lisboa Portugal; ^3^ Department of Epidemiology National Institute of Health Doutor Ricardo Jorge Lisboa Portugal; ^4^ Comprehensive Health Research Center, NOVA Medical School Universidade NOVA de Lisboa Lisboa Portugal; ^5^ National School of Public Health Universidade NOVA de Lisboa Lisboa Portugal; ^6^ Department of Infectious Diseases Epidemiology Bernhard Nocht Institute for Tropical Medicine Hamburg Germany

**Keywords:** children, Europe, molecular characterization, respiratory viruses, RSV, surveillance

## Abstract

**Background:**

Respiratory syncytial virus (RSV) is the leading cause of acute respiratory infection (ARI) in young children, but its genetic diversity requires ongoing surveillance.

**Methods:**

From 2021 to 2023, a total of 619 and 94 RSV‐positive samples from the National Respiratory Syncytial Virus Surveillance Network (VigiRSV) and the Sentinel Influenza and other respiratory viruses surveillance network (Sentinel ISN), respectively, were analysed. The RSV A and RSV B typing was assessed by a multiplex real‐time RT‐PCR. Sanger sequencing was performed on a subset of samples (*n* = 495). Phylogenetic analysis was carried out on partial glycoprotein G sequences. Clinical and epidemiological data were compared through Pearson Chi‐Square tests.

**Results:**

RSV Subgroup A was more prevalent (53.5%, 85/159) in the 2021/2022 season, whereas in the 2022/2023 season, it was RSV Subgroup B (82.1%, 435/530) in both networks. RSV A strains in VigiRSV clustered mainly to a.d.1 (39.0%, 39/100), whereas in Sentinel ISN, they clustered in a.d.5 (30.0%, 3/10). RSV Type B clustered mainly to B.D.E.1 (96.6%, 372/385) in both networks. All lineages cocirculated during the study period and in both surveillance networks. Regional clusters were identified for both subgroups.

**Conclusions:**

This study provides new insights into RSV genetic variability in Portugal, namely, the cocirculation of lineages and intravariability among lineages within both subgroups during the study period and in all Portuguese regions. However, our study is based on partial sequencing of the G gene, and because of this limitation, our results should be considered with great caution.

## 1 Introduction

The human respiratory syncytial virus (RSV) is a respiratory pathogen recognized as a leading cause of acute respiratory infection (ARI) in young children, the elderly and immunocompromised individuals [1, 2]. Infants and children under the age of 5 years old are particularly susceptible to severe RSV infection, commonly presenting with bronchiolitis and pneumonia [3, 4]. According to a global review of RSV burden, an estimated 33 million RSV‐associated acute lower respiratory infection episodes occurred in 2019, resulting in 26,300 RSV in‐hospital deaths, and around 102,000 RSV overall deaths in children under 5 years old [5].

RSV is a member of the *Pneumoviridae* family and the Orthopneumovirus genus [6]. This virus is enveloped and contains a single‐stranded nonsegmented negative‐sense RNA of approximately 15.2 kb in length and has 10 genes, which encode for 11 separate proteins [7, 8, 9], including three transmembrane glycoproteins that are present in the viral envelope (small hydrophobic [SH], attachment [G] and fusion [F]) [10]. The G and F proteins are the major targets of the host humoral response and are potential candidates for subunit vaccines [11, 12, 13, 14, 15, 16, 17, 18].

RSV is divided into two subgroups, A and B, even so containing one serosubtype [12, 19], which cocirculate during epidemic seasons with one of them usually predominating [8, 13]. RSV, like other RNA viruses, has a high mutation rate, leading to alterations in sequence and antigenicity, especially in the G protein [12]. Although the F protein does not show much variation, the G protein exhibits greater genetic and antigenic variability among RSV strains; historically, the lineage definition is based on the genetic characteristics of the second variable region of this protein [14, 15]. However, current definitions by the *RSV Genotyping Consensus Consortium* are based on complete genomes due to the limitations of partial genome analysis [14, 20].

Due to the lack of consensus regarding the lineage classification [20], a new system was proposed in 2024 by the *RSV Genotyping Consensus Consortium* (RGCC) [14], which is applied by the Nextclade platform [21]. A unified nomenclature and phylogenetic classification are indispensable for investigating the correlation between lineage and disease severity or the geographical distribution of the virus, which could be facilitated by data from surveillance [14, 18].

Despite the significant public health impact of RSV, only the monoclonal antibody Palivizumab and the viral replication inhibitor Ribavirin were available for the prevention and treatment of RSV for a long time. Most recently, three vaccines (two for the elderly and one for pregnant women) targeting RSV F protein, as well as the monoclonal antibody Nirsevimab, have been approved in 2023, enhancing the available options to prevent severe RSV disease [22, 23, 24]. However, potential amino acid changes on the RSV genome may have a possible negative effect on the performance of RSV therapeutics [25]. In this sense, it is crucial to develop more studies about RSV and to establish molecular RSV surveillance systems to monitor the genetic diversity and emerging resistance to RSV preventive therapeutics [18, 25].

Aiming to standardize RSV surveillance, the World Health Organization (WHO) established, in 2016, an RSV surveillance pilot study based on the Global Influenza Surveillance and Response System (GISRS) [26, 27]. Several recommendations came from this pilot project, including prioritizing RSV surveillance in children under 2 years old and focusing on severe RSV disease that requires hospitalization [27]. Therefore, a Portuguese RSV sentinel network was established in 2021 to monitor RSV infections in hospitalized children below 2 years old [26]. In addition, since 2010, the surveillance of RSV among ambulatory settings has been made by General Practitioners through the Sentinel network into the Portuguese Influenza Surveillance System (ISN) during influenza season [20, 28].

Few studies on RSV have been conducted in Portugal, with only one focusing on epidemiology and genetic variability, and using data up to 2018 [28]. Therefore, there is an urgent need to expand these studies, which could help to identify the genetic and epidemiological diversity of RSV in Portugal. The objective of this study was to assess the genetic diversity of respiratory syncytial virus (RSV) strains identified in Portugal between 2021 and 2023, comparing RSV strains from hospitalized paediatric patients up to 2 years with those detected in community patients in primary healthcare settings of all age groups.

## 2 Methods

### 2.1 Setting

The Sentinel Influenza and other respiratory viruses surveillance network (Sentinel ISN) weekly select and collect nasopharyngeal swabs from acute respiratory infections (ARI) for respiratory virus diagnosis at INSA [29]. ARI cases reported were validated according to the World Health Organization definition criteria [30]: (a) acute sudden onset of symptoms and (b) at least one of the following respiratory symptoms: shortness of breath or cough, sore throat and/or coryza.

The National Respiratory Syncytial Virus Surveillance Network (VigiRSV) comprises 20 public and private hospitals from the five mainland regional health administrations (Norte, Centro, Lisboa e Vale do Tejo, Alentejo and Algarve) and Região Autónoma da Madeira, of Portugal. Hospitals belonging to this network report and test ARI cases in children < 2 years old, hospitalized for at least 24 h [26].

### 2.2 Study Population and Sample Collection

We conducted the study from October 2021 to April 2023. Samples were collected from two networks: (a) hospitalized children up to 2 years old reported by the VigiRSV and (b) all age ARI community patients reported by the Sentinel ISN.

RSV‐positive samples from VigiRSV were sent to the National Reference Laboratory for Influenza and Other Respiratory Viruses (LNRVG) at the National Institute of Health Doutor Ricardo Jorge (INSA). Regarding Sentinel ISN, all samples from recruited patients were collected by general practitioners and sent to LNRVG for respiratory viruses laboratory diagnosis. These samples included nasopharyngeal and/or oropharyngeal swabs (from VigiRSV and Sentinel ISN), nasopharyngeal aspirates and bronchoalveolar lavages (from VigiRSV).

### 2.3 RSV Molecular Detection and Subtyping

Samples originated from Sentinel ISN were tested for RSV using the Allplex SARS‐CoV‐2/FluA/FluB/RSV (Seegene Inc., Seoul, South Korea) kit. All RSV‐positive samples were subgrouped using the Allplex Respiratory Panels 1 (Seegene Inc., Seoul, South Korea) kit at LNRVG.

Samples from VigiRSV were tested at the hospital laboratory using molecular methods (RT‐PCR). RSV‐positive samples were sent to LNRVG for subtyping and genetic characterization. Subtyping was performed using a multiplex real‐time RT‐PCR assay adapted from a previous protocol described by Wang et al. [31].

### 2.4 Genetic Sequencing and Characterization

RSV strain characterization was conducted on all positive samples with a cycle threshold (*Ct* value) ≤ 25, using Sanger sequencing, targeting the Hypervariable Region 2 (HVR2) of glycoprotein G using a nested RT‐PCR, adapted from previously published protocols [30, 32]. In the first round of amplification, RSV3 (GGCAATGATAATCTCAAC) and F164 (GTTATGACACTGGTATACCAACC) primers were used, whereas in the second round, OGCH496 + (GATGATTACCATTTTGAAGTGTTCA) and F1 (CAACTCCATTGTTATTTGCC) primers were selected. The approximately 500‐bp fragments were visualized by the QIAxcel Advanced system (QIAGEN).

### 2.5 Phylogenetic Analysis

Sequence analysis was carried out using MEGA 7.0 software [33]. Nucleotide sequences were aligned with the ClustalW method in MAFFT Version 7 [34, 35] and maximum likelihood phylogenetic trees were generated with IQ‐TREE v3.0.1, using ModelFinder to find the best nucleotide substitution model [36, 37]. The reliability of sequence clusters was analysed with SH‐aLRT and UFBoot2 (with 1000 replicates) [38]. The phylogenetic trees were visualized and coloured in iTOL v6 [39]. All reference strains (RSV A and RSV B) from the RGCC GitHub page [40] (https://github.com/rsv‐lineages/lineage‐designation‐A and https://github.com/rsv‐lineages/lineage‐designation‐B) were used for the construction of the phylogenetic trees. The classification of these references and our sequences was further validated using Nextclade [21], which follows the lineage definitions proposed by the RGCC [14]. All references are listed in Tables S4 and S5, and accession numbers of our sequences are listed in Table S6.

### 2.6 Clinical and Epidemiological Data Analysis

VigiRSV samples were sent from five health regions of Portugal mainland (Alentejo, Algarve, Centro, Lisboa e Vale do Tejo, Norte), data on sex, age group (0–3, 4–6, 7–12 or 13–23 months) and clinical presentations with both networks were collected (cough, sore throat, fever and shortness of breath). These data were filled in an online questionnaire through the REDCap [41] (Research Electronic Data Capture) platform or on paper by the physician responsible for the surveillance in each participating hospital.

The data obtained from community cases through Sentinel ISN included the sampling location, gender and age/date of birth (0–2, 3–17, 18–64 or > 65 years) and clinical presentations in common with both networks (cough, sore throat, fever and shortness of breath). This information was recorded in paper questionnaires by the Sentinel General Practitioner (GP) and transferred to REDCap by the INSA team.

### 2.7 Statistical Analysis

Pearson Chi‐square tests were used to compare the surveillance networks and to compare the subgroups in each network. Tests with *p* value < 0.05 were considered statistically significant. Analyses were performed using IBM SPSS Statistics, Version 27.

### 2.8 Ethical Considerations

This study was conducted in strict accordance to protect patient privacy and anonymity. The Health Ethics Committee of the National Institute of Health Doutor Ricardo Jorge (INSA) approved surveillance activities performed under the VigiRSV network with the reference 19/2021, and the Sentinel ISN network with Reference 65/2022. Furthermore, the project where this paper is included was approved by the Ethical Committee of NMS/FCM‐UNL (Process No. 63/2023/CEFCM).

## 3 Results

### 3.1 General Characterization of the Study Population

From October 2021 to April 2023, a total of 1569 (Table S1) and 1594 (Table S2) respiratory infections from the VigiRSV and the Sentinel ISN, respectively, were reported. From these, 986 were laboratory‐confirmed RSV cases (892 from VigiRSV and 94 from Sentinel ISN). For RSV characterization, 713 (72.3%) RSV‐positive samples were sent to LNRVG at INSA (619 from VigiRSV and 94 from Sentinel ISN).

The Lisboa and Vale do Tejo region had more laboratory‐confirmed RSV‐positive cases in the VigiRSV network, whereas the Norte region had more laboratory‐confirmed RSV‐positive cases in the Sentinel ISN network. Children aged 3–5 months and adults aged 18–64 years were the most affected groups in the VigiRSV and Sentinel ISN, respectively. In VigiRSV, the higher predominance was observed in males, whereas in Sentinel ISN, it was in females (Tables S1 and S2).

The overall RSV positivity rate in both networks was 56.9% and 5.9% for VigiRSV and Sentinel ISN, respectively. The positivity rate in Algarve (97.0%, 64/66 for VigiRSV; 10.0%, 3/30 for Sentinel ISN) was the highest in both networks (Tables S1 and S2). In the VigiRSV network, the highest positivity rate was in children from 3 to 5 months old, comprising 64.3%. In the Sentinel ISN, the most represented age group in terms of positivity was below 3 years old, accounting for 12.5% (Tables S1 and S2). The higher positivity rate was observed in females in both networks, with 57.8% and 6.1% for VigiRSV and Sentinel ISN, respectively (Tables S1 and S2).

The clinical manifestations for the 713 RSV‐positive samples analysed are described in Tables S3 and Table 1. Among the RSV cases detected through the VigiRSV network, we had 7.8% or 10.2% of cases that reported symptoms (Table S3). Within them, cough was the most frequently reported symptom (45/48) (Table 1). In contrast, cases identified through the Sentinel ISN network provided more comprehensive clinical information (93.4% or 94.7% of all RSV‐positive cases from this network) (Table S3), with cough reported in 75 of 88 cases (85.2%) and sore throat in 53 cases (60.2%), among those with available clinical data (Table 1). In both surveillance networks, fever was absent in most of the cases (30/48 [62.5%] in VigiRSV and 52/89 [58.4%] in Sentinel ISN) (Table 1).

**TABLE 1:** Overview of the clinical presentations from all RSV‐positive samples received at LNRVG of INSA that reported the presence of symptoms divided into the age group through the National Respiratory Syncytial Virus Surveillance Network (VigiRSV) and the Sentinel Influenza and other respiratory viruses surveillance network (Sentinel ISN).

		Cough	Sore throat	Fever	Shortness of breath
		*n* (%)	*n* (%)	*n* (%)	*n* (%)
	Total	45 (93.8%)	NA	18 (37.8%)	0 (0%)
	Age group				
VigiRSV	< 3 months	21 (46.7%)	NA	5 (27.8%)	0 (0%)
	3–5 months	10 (22.2%)	NA	5 (27.8%)	0 (0%)
	6–11 months	9 (20.0%)	NA	6 (33.3%)	0 (0%)
	12–23 months	5 (11.1%)	NA	2 (11.1%)	0 (0%)
	Total	75 (85.2%)	53 (60.2%)	37 (41.6%)	10 (11.4%)
	Age group				
Sentinel ISN	< 3 years	2 (2.7%)	NA	1 (2.7%)	1 (10.0%)
	3–17 years	10 (13.3%)	10 (18.9%)	9 (24.3%)	3 (30.0%)
	18–64 years	41 (54.7%)	35 (66.0%)	19 (51.4%)	5 (50.0%)
	≥ 65 years	22 (29.3%)	8 (15.1%)	8 (21.6%)	1 (10.0%)

Abbreviation: NA, not applicable.

**FIGURE 1:**
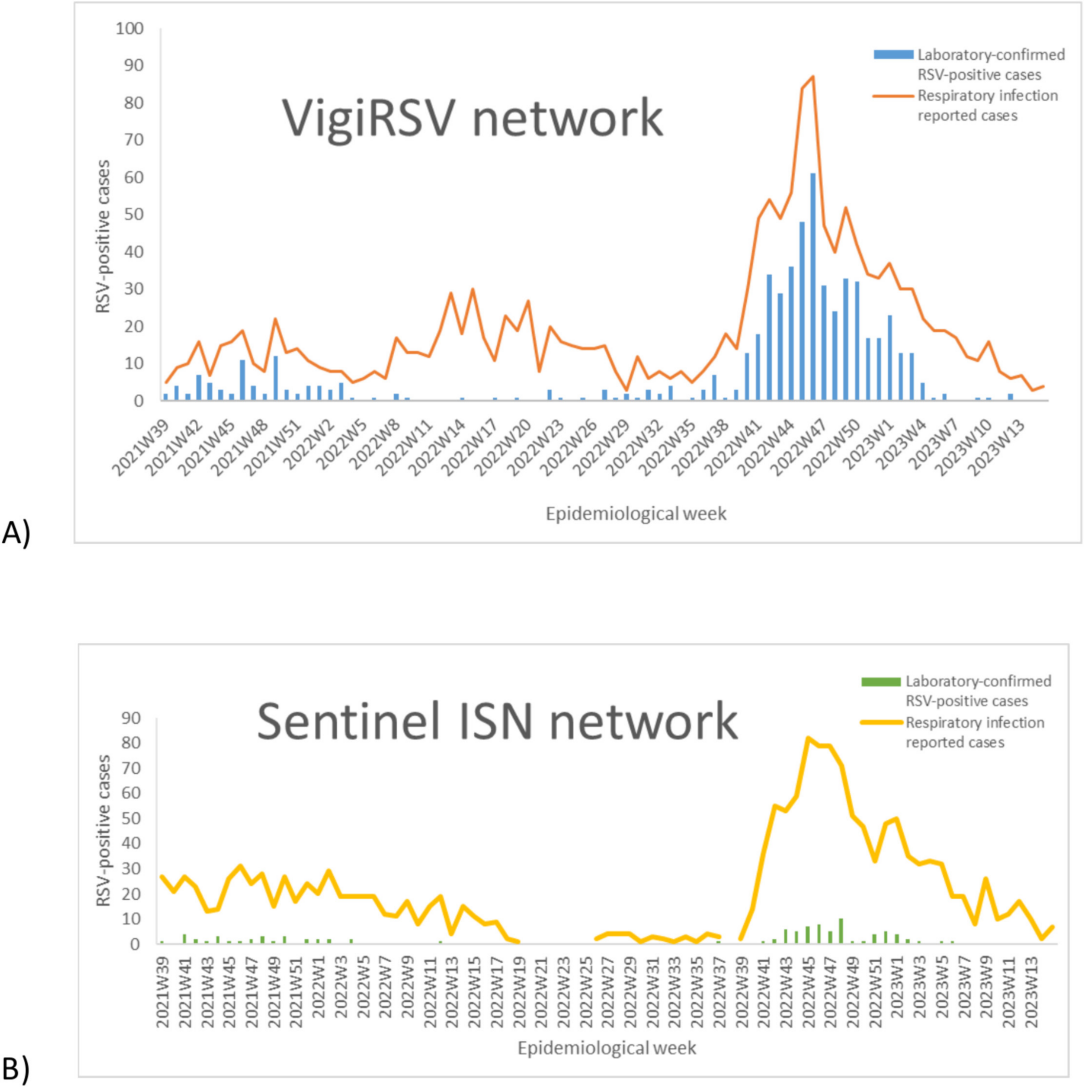
Weekly distribution of acute respiratory infection (ARI, WHO definition) reported cases in the VigiRSV (A) and in the Sentinel ISN (B) networks, from the week 2021/39 (October 2021) to week 2023/15 (April 2023), in Portugal (orange/yellow line). The blue/green bars correspond to the laboratory‐confirmed Respiratory Syncytial Virus (RSV) positive cases in the respective networks.

We observed in both networks a low circulation of RSV in the 2021/2022 season, with a significant increase in the 2022/2023 season, particularly from October 2022 (2022W39) to January 2023 (2023W04) (Figure 1A,B).

### 3.2 RSV Typing

From 713 RSV‐positive samples received in the LNRVG, 689 were subgrouped (595 from the VigiRSV network and 94 from the Sentinel ISN network). For 24 RSV‐positive samples from the VigiRSV (4 in the 2021/22 season and 20 in the period 2022/23), we were not able to identify the subgroup due to its high CT value (≥ 40).

During the 2021/2022 season, RSV A was presented in 53.5% (85/159) of the received samples, and, in the 2022/2023 season, RSV subgroup B (RSV B) was the most prevalent subgroup, detected in 82.1% (435/530) of the samples (Table 2).

**TABLE 2:** RSV‐positive samples subgrouped in the National Reference Laboratory for Influenza and Other Respiratory Virus (LNRVG) of INSA, through the National Respiratory Syncytial Virus Surveillance Network (VigiRSV) and the Sentinel Influenza and other respiratory viruses surveillance network (Sentinel ISN).

		2021/2022 season				2022/2023 season		
		RSV A	RSV B			RSV A	RSV B	
	*N*	*n* (%)	*n* (%)	*p* value^a^	*N*	*n* (%)	*n* (%)	*p* value^a^
Total	159	85 (53.5%)	74		530	95	435 (82.1%)	
VigiRSV	129	69 (53.5%)	60	0.988	466	87	379 (81.3%)	0.228
Sentinel ISN	30	16 (53.3%)	14		64	8	56 (87.5%)	

^a^ Chi test was used to compare the subgroups between the surveillance systems. Tests with *p* value < 0.05 were considered statistically significant.

In the Sentinel ISN network, just like in the VigiRSV network, RSV A presented 53.3% (16/30) and 53.5% (69/129), respectively, of predominance over RSV B in the 2021/2022 season. In the following season, and just like the previous season, but with a subgroup shift, VigiRSV and Sentinel ISN presented similar proportions (81.3%, 379/466, and 87.5%, 56/64, respectively) for RSV B. No significant difference in subgroup prevalence was observed between the two networks during both seasons (*p* = 0.988 and *p* = 0.228, respectively).

### 3.3 Demographic and Geographic Characteristics of the RSV‐Positive Samples According to Subgroup

Of the 689 RSV‐positive subgrouped samples, 71.8% (495/689) were selected for genetic characterization because they presented a *Ct* value of ≤ 25. Of these, 71.8% (427/595) were from VigiRSV and 72.3% (68/94) from Sentinel ISN. Demographic and geographic characteristics of the characterized RSV‐positive samples for VigiRSV were described in Table 3. In this network, RSV B was the predominant subgroup characterized, with 327 in comparison to 100 RSV A. There are significant differences (*p* = 0.004) when comparing subgroups between regions, but they are not significant between age groups (*p* = 0.441) and gender (*p* = 0.530).

**TABLE 3:** Overview on the demographic and geographic characteristics from all characterized RSV‐positive samples divided into subgroups through the National Respiratory Syncytial Virus Surveillance Network (VigiRSV) and the Sentinel Influenza and other respiratory viruses surveillance network (Sentinel ISN).

		Sentinel ISN network				VigiRSV network		
		RSV A	RSV B	*p* value^a^		RSV A	RSV B	*p* value^a^
	*N*	*n* (%)	*n* (%)		*N*	*n* (%)	*n* (%)	
Total	68	10	58		427	100	327	
Regions				0.503				0.004
Alentejo		1 (10.0%)	4 (7.0%)			12 (12.0%)	27 (8.3%)	
Algarve		0 (0%)	3 (5.2%)			2 (2.0%)	48 (14.7%)	
Centro		3 (30.0%)	9 (15.5%)			10 (10.0%)	17 (5.2%)	
Lisboa e Vale do Tejo		4 (40.0%)	18 (31.0%)			48 (48.0%)	159 (48.6%)	
Norte		2 (20.0%)	24 (41.4%)			28 (28.0%)	76 (23.2%)	
Age group				0.672				0.441
< 3 months		NA	NA			60 (60.0%)	184 (56.3%)	
3–5 months		NA	NA			20 (20.0%)	66 (20.2%)	
6–11 months		NA	NA			14 (14.0%)	46 (14.1%)	
12–23 months		NA	NA			6 (6.0%)	31 (9.5%)	
< 3 years		0 (0%)	2 (3.4%)			NA	NA	
3–17 years		2 (20.0%)	6 (10,3%)			NA	NA	
18–64 years		5 (50.0%)	33 (56.9%)			NA	NA	
≥ 65 years		3 (30.0%)	17 (29.3%)			NA	NA	
Gender				0.114				0.530
Male		1 (10.0%)	18 (31.0%)			58 (58.0%)	178 (54.4%)	
Female		9 (90.0%)	37 (63.8%)			42 (42.0%)	149 (45.6%)	

Abbreviation: NA, not applicable.

^a^ Chi test was used to compare the subgroups between the regions, age groups and genders. Tests with *p* value < 0.05 were considered statistically significant.

In the Sentinel ISN network (Table 3), RSV B was also the most predominant subgroup characterized, with the identification of 58 cases in comparison to 10 cases of RSV A. There are no differences when comparing subgroups among regions, age groups or gender among the RSV cases detected through this network (*p* = 0.503, *p* = 0.672 and *p* = 0.114, respectively).

### 3.4 Phylogenetic Characterization

Regarding the phylogenetic characterization, the 110 RSV A characterized viruses (100 from VigiRSV and 10 from Sentinel ISN networks) were clustered into nine lineages (Table 4 and Figure 3): a.d.1, a.d.1.7, a.d.3, a.d.3.1, a.d.3.3, a.d.4, a.d.5, A.D.5.1 and A.D.5.2. When comparing the two surveillance networks, there are no significant differences (*p* = 0.169) between the distribution of the lineages (Table 4). Within the VigiRSV network, the a.d.1 lineage was the largest group (39.0%, 39/100). Among the Sentinel ISN network, Lineage a.d.5 was the largest group (30.0%, 3/10).

**TABLE 4:** Distribution of the lineage in each subgroup RSV A and RSV B of the National Respiratory Syncytial Virus Surveillance Network (VigiRSV) and the Sentinel Influenza and other respiratory viruses surveillance network (Sentinel ISN).

		VigiRSV network	Sentinel ISS network	*p* value^a^
	*N*	*n* (%)	*n* (%)	
Total	110	100 (90.9%)	10 (9.1%)	
RSV A				0.169
A.D.1		39 (39.0%)	1 (10.0%)	
A.D.1.7		1 (1.0%)	0 (0%)	
A.D.3		28 (28.0%)	2 (20.0%)	
A.D.3.1		4 (4.0%)	2 (20.0%)	
A.D.3.3		0 (0%)	1 (10.0%)	
A.D.4		1 (1.0%)	0 (0%)	
A.D.5		21 (21.0%)	3 (30.0%)	
A.D.5.1		1 (1.0%)	0 (0%)	
A.D.5.2		5 (5.0%)	1 (10.0%)	
Total	385	327 (84.9%)	58 (15.1%)	
RSV B				0.955
B.D.4.1.1		6 (1.8%)	1 (1.7%)	
B.D.4.1.3		2 (0.6%)	0 (0%)	
B.D.E.1		316 (96.6%)	56 (96.6%)	
B.D.E.1.5		3 (0.9%)	0 (0%)	
B.D.E.5		0 (0%)	1 (1.7%)	

^a^ Chi‐test was used to compare the surveillance systems between the lineages. Tests with *p* value < 0.05 were considered statistically significant.

Relative to RSV B strains, phylogenetic analysis revealed that 385 sequences (327 from VigiRSV and 58 from ISN networks) clustered into five lineages (Table 4 and Figure 4): B.D.4.1.1, B.D.4.1.3, B.D.E.1, B.D.E.1.5 and B.D.E.5. There are no significant differences (*p* = 0.955) between the distribution of the lineages through both surveillance systems (Table 4). The B.D.E.1 lineage was the largest lineage among cases from the VigiRSV network (96.6%, 316/327) and the Sentinel ISN (96.6%, 56/58).

Comparing the lineages with the age groups across the VigiRSV network, in Table 5, we observed that the VigiRSV network's largest group (a.d.1 lineage) was predominant in children under 3 months of age (43.6%, 17/39). Among the Sentinel ISN network, the a.d.5 lineage was predominant in adults over 65 years old (66.7%, 2/3). Regarding RSV B, the B.D.E.1 lineage was predominant in all age groups from the VigiRSV network (74.0%, 319/427) and the Sentinel ISN (82.4%, 56/68).

**TABLE 5:** Distribution of the genetic lineages according to age‐matched groups through the National Respiratory Syncytial Virus Surveillance Network (VigiRSV) and the Sentinel Influenza and other respiratory viruses surveillance network (Sentinel ISN).

			RSV A									RSV B				
			A.D.1	A.D.1.7	A.D.3	A.D.3.1	A.D.3.3	A.D.4	A.D.5	A.D.5.1	A.D.5.2	B.D.4.1.1	B.D.4.1.3	B.D.E.1	B.D.E.1.5	B.D.E.5
		*N*	*n* (%)	*n* (%)	*n* (%)	*n* (%)	*n* (%)	*n* (%)	*n* (%)	*n* (%)	*n* (%)	*n* (%)	*n* (%)	*n* (%)	*n* (%)	*n* (%)
VigiRSV	Total	427	39 (9.1%)	1 (0.2%)	28 (6.6%)	4 (0.9%)	0 (0%)	1 (0.2%)	21 (4.9%)	1 (0.2%)	5 (1.2%)	6 (1.4%)	2 (0.5%)	316 (74.0%)	3 (0.7%)	0 (0%)
	Age group															
	< 3 months		17 (43.6%)	1 (100%)	21 (75.0%)	4 (100%)	0 (0%)	0 (0%)	12 (57.1%)	1 (100%)	4 (80.0%)	4 (66.7%)	1 (50.0%)	176 (55.7%)	3 (100%)	0 (0%)
	3–5 months		9 (23.1%)	0 (0%)	5 (17.9%)	0 (0%)	0 (0%)	1 (100%)	4 (19.0%)	0 (0%)	1 (20.0%)	0 (0%)	0 (0%)	66 (20.9%)	0 (0%)	0 (0%)
	6–11 months		9 (23.1%)	0 (0%)	2 (7.1%)	0 (0%)	0 (0%)	0 (0%)	3 (14.3%)	0 (0%)	0 (0%)	2 (33.3%)	0 (0%)	44 (13.9%)	0 (0%)	0 (0%)
	12–23 months		4 (10.3%)	0 (0%)	0 (0%)	0 (0%)	0 (0%)	0 (0%)	2 (9.5%)	0 (0%)	0 (0%)	0 (0%)	1 (50.0%)	30 (9.5%)	0 (0%)	0 (0%)
Sentinel ISN	Total	68	1 (1.5%)	0 (0%)	2 (2.9%)	2 (2.9%)	1 (1.5%)	0 (0%)	3 (4.4%)	0 (0%)	1 (1.5%)	1 (1.5%)	0 (0%)	56 (82.4%)	0 (0%)	1 (1.5%)
	Age group															
	< 3 years		0 (0%)	0 (0%)	0 (0%)	0 (0%)	0 (0%)	0 (0%)	0 (0%)	0 (0%)	0 (0%)	0 (0%)	0 (0%)	2 (3.6%)	0 (0%)	0 (0%)
	3–17 years		0 (0%)	0 (0%)	0 (0%)	2 (100%)	0 (0%)	0 (0%)	0 (0%)	0 (0%)	0 (0%)	0 (0%)	0 (0%)	6 (10.7%)	0 (0%)	0 (0%)
	18–64 years		1 (100%)	0 (0%)	1 (50.0%)	0 (0%)	1 (100%)	0 (0%)	1 (33.3%)	0 (0%)	1 (100%)	0 (0%)	0 (0%)	33 (58.9%)	0 (0%)	0 (0%)
	≥ 65 years		0 (0%)	0 (0%)	1 (50.0%)	0 (0%)	0 (0%)	0 (0%)	2 (66.7%)	0 (0%)	0 (0%)	1 (100%)	0 (0%)	15 (26.8%)	0 (0%)	1 (100%)

**FIGURE 2:**
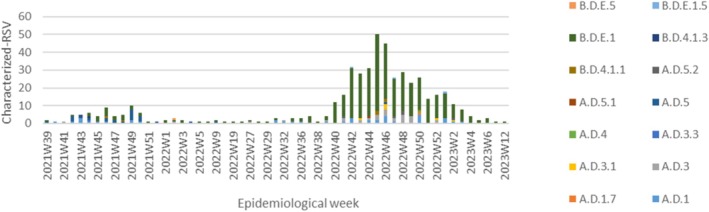
Weekly distribution of characterized‐RSV, from week 2021/39 (October 2021) to week 2023/12 (April 2023), in Portugal. The coloured bars correspond to the lineages in circulation by week.

**FIGURE 3:**
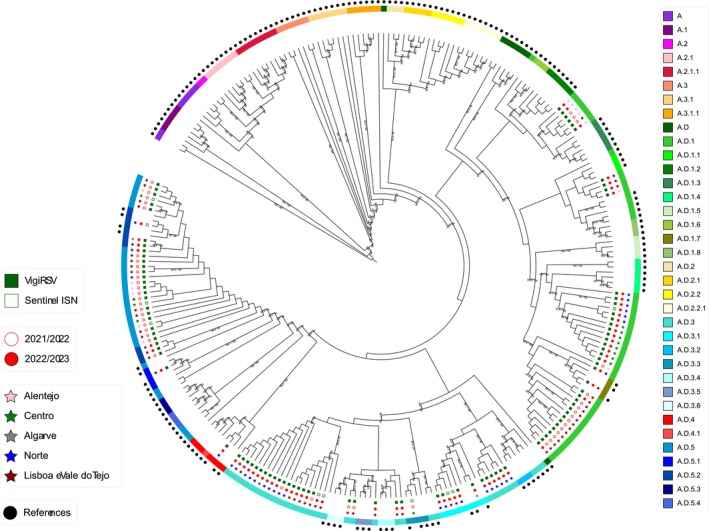
Phylogenetic analysis of RSV A circulating in Portugal, from October 2021 to April 2023. The C‐terminal second hypervariable region (HVR2) of the G gene sequences from Portugal and reference strain sequences were used to generate a phylogenetic tree using the maximum likelihood method. The reliability of sequence clusters was evaluated with SH‐aLRT (1000 replicates) and UFBoot2 (1000 replicates). Only values of SH‐aLRT ≥ 80% and UFBoot2 ≥ 90% were represented at the branch nodes. Colour‐coded information on each RSV includes the surveillance system which each sample came from (Column 1), the season of the sample collection (Column 2), region (Column 3) and lineage (Column 4). Reference sequences are indicated in black circles and are listed in Table S4. The phylogeny distribution can be visualized at https://itol.embl.de/export/94632137543561750344481 using iTOL v6 (https://itol.embl.de/).

**FIGURE 4:**
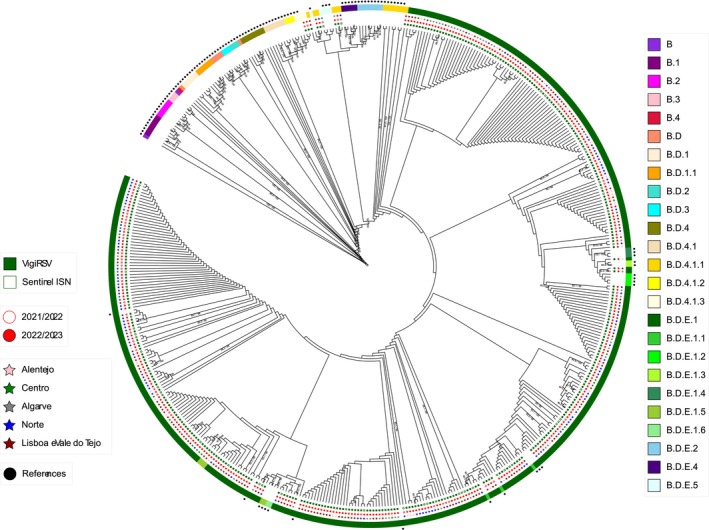
Phylogenetic analysis of RSV B circulating in Portugal, from October 2021 to April 2023. The C‐terminal second hypervariable region (HVR2) of the G gene sequences from Portugal and reference strain sequences were used to generate a phylogenetic tree using the maximum likelihood method. The reliability of sequence clusters was evaluated with SH‐aLRT (1000 replicates) and UFBoot2 (1000 replicates). Only values of SH‐aLRT ≥ 80% and UFBoot2 ≥ 90% were represented at the branch nodes. Colour‐coded information on each RSV includes the surveillance system which each sample came from (Column 1), the season of the sample collection (Column 2), region (Column 3) and lineage (Column 4). Reference sequences are indicated in black circles and are listed in Table S5. The phylogeny distribution can be visualized at https://itol.embl.de/export/946321375269701750337992 using iTOL v6 (https://itol.embl.de/).

We observed in Figure 2 that RSV A and RSV B lineages cocirculated during the study period, showing a significant genetic variability of circulating lineages from October 2022 (2021W39) to April 2023 (2023W12). Despite the 2021/2022 outbreak, which did not follow a typical seasonal distribution, the a.d.5 lineage presented the highest frequency, whereas in the peak of the 2022/23 season, it was Lineage B.D.E.1. This genetic diversity is shown in Figures 3 and 4, along with the geographic distribution, the surveillance network and the collection season of each characterized RSV.

Another key point observed was the detection of regional clusters within each lineage for the respective regions. For RSV A (Figure 3), during the 2021/2022 season, Lineages a.d.1 and A.D.5 were more frequent. Most clusters detected in the Lineage a.d.1 were from the Lisboa e Vale do Tejo region, whereas the Alentejo and Centro regions exhibited clusters within the a.d.5 lineage. In the subsequent season, Lineages a.d.1 and A.D.3 were revealed to be more frequent. Additionally, multiple clusters in the a.d.1 and A.D.3 lineages were detected in the Norte region, whereas the Lisboa e Vale do Tejo region showed clusters in the a.d.3 lineage. Additionally, the Centro and Alentejo regions revealed more clusters in the a.d.1 lineage. RSV A phylogenetic trees in Figures S1 and S2 supported these different genetic clusters, with the Portuguese sequences challenging against all European sequences of those seasons. In the RSV B phylogenetic tree (Figure 4), clusters in the B.D.E.1 lineage were detected in all regions during both seasons and were supported using the same approach mentioned for RSV A (Figures S3 and S4).

## 4 Discussion

This study had the advantage of including a large study population from two different settings during two consecutive seasons in Portugal. From the obtained data, it was possible to compile a comprehensive overview of RSV epidemiology and circulation patterns nationwide, from a recent period. Overall, in this study, we showed a cocirculation of lineages within both subgroups (RSV A and RSV B) during the study period that contributes to RSV knowledge in Portugal.

We detected a low circulation of RSV in the 2021/2022 season, with an increase in autumn/winter months in the 2022/2023 season. The circulation peak during this season was noted 2 weeks earlier in the VigiRSV network, compared to the Sentinel ISN, and in contrast to other European countries [42]. During the 2021/2022 period, the RSV epidemic overlapped with the COVID‐19 pandemic, resulting in a very reduced RSV transmission [43]. The lower proportion of RSV cases observed in this study in 2021/2022 may be associated with the anti‐COVID‐19 measures to lower the risk of COVID‐19 transmission that were still in place at that time, which were consistent with reports from other territories [44, 45]. Because of the low RSV exposure in the 2021/2022 season, there was probably, linked to a significant reduction of immunity in the population, causing an early start of the 2022/2023 epidemic, registered in Portugal and many other European countries [42, 46].

The positivity rate of RSV is higher in children from 3 to 5 months old (Table S1). In Sentinel ISN, the highest positivity rate of the RSV cases was in children below 3 years old (Table S2) and was very similar to another risk age group, the > 65 years old. Our observations in the Sentinel ISN network are in line with many reports that showed that RSV infections are much more frequent in children aged under 2 years, decreasing with an increase in age due to the development of immunity [47] and appearing again from 65 years old and over [10]. However, it is important to note that the VigiRSV population is children below 2 years old, a population at high risk, and so they will go directly to hospitals, whereas the majority of the Sentinel ISN population belongs to the age group of 18–64 years, a population where the infection is generally mild [48, 49], which could explain the lower number reported of positive RSV cases in the Sentinel ISN network.

This work demonstrated the importance of establishing different surveillance systems, considering the risk groups and characteristics of the target pathogen. VigiRSV was set up to monitor RSV‐hospitalized cases in children up to 2 years old. In our study, this network presented a positivity rate 10 times higher than the community surveillance system. We also presented an unusual positivity rate in the Algarve compared to the other regions, and this could be explained by a limitation in the VigiRSV network with the ARI reported cases. Although we request a report of all ARI cases, we are verifying a trend of the hospitals reporting preferentially RSV‐positive cases.

During the study period, hospitals from Lisboa e Vale do Tejo reported more RSV‐positive cases to LNRVG through the VigiRSV network, whereas in the Sentinel ISN network were primary care centres from the Norte region. The difference in the proportion of RSV‐positive cases is following the number of reported cases in the Sentinel ISN. This was not observed in VigiRSV, in which the region with the highest number of reported ARI cases (Norte) turns out to be the second region with the highest number of laboratory‐confirmed RSV‐positive cases, behind Lisboa e Vale do Tejo. In this way, there was a difference in positive patterns among the networks. The surveillance systems are assembled to be representative of the country; however, it always depends on the participation of the doctors, and this may explain the results, but these data must be analysed with caution. We hypothesized that the Norte health centres are more participative than the others, with a significant difference in the Sentinel ISN than in the VigiRSV.

On clinical signs and symptoms of RSV cases detected through the VigiRSV network, we observed that cough was present in the majority among the cases with informed symptoms, which was consistent with data reported worldwide [50]. More information was obtained from the cases of the Sentinel ISN network, and we observed a higher number of cases with cough and sore throat. From the VigiRSV network, data on signs and symptoms were less complete than Sentinel ISN. These differences can be justified due to the differences between the networks and the participation of the doctors and young children in describing their signs and symptoms. Unfortunately, our general sample size is too small to find more correlations among the clinical presentations.

RSV Subgroup A was the most prevalent subgroup detected in the 2021/2022 season in both networks. In the 2022/23 season, RSV Subgroup B surpassed RSV A and became the most prevalent subgroup detected in both networks, which was consistent with the seasonal circulation reported by other European countries [46].

Phylogenetic analysis of RSV has been done in many parts of the world. However, comprehensive genomic analyses at high levels are only made based on available whole‐genome data [14]. A.1 and A.2 lineages from RSV A were described as extinct [14]. By 2011, A. D and descendant lineages continue to circulate, and since 2015, only these lineages, with the 72‐nt duplication in the G gene, have been observed [14]. In RSV Subgroup B, although the 60‐nt duplication in the G gene, presented in the B. D lineage, and originally observed in 1999, only by 2009 these lineages and their lineage representatives were in circulation, and since 2017, only B.D.4 and all descendants were detected [14].

Our phylogenetic analysis of the HVR2 region of glycoprotein G of RSV B showed that the B.D.E.1 was the predominant lineage in both networks (hospitalized and community), consistent with data reported worldwide [51]. The B.D.E.1, a lineage identified only in the postpandemic period [52] was considered a descendant of the B.D.4.1.1 lineage [53]. Within the B.D.E.1 lineage, it was described that five lineage markers in this glycoprotein were prominent: S100G, K256N, I268T, S275P and Y285H [50]. In our study, only 3.4% (13/385) of RSV B belonged to other lineages (B.D.4.1.1, B.D.4.1.3, B.D.E.1.5 and B.D.E.5). Relatively to RSV A, in the VigiRSV network, the majority of characterized RSV belonged to the a.d.1 lineage, whereas in Sentinel ISN, Lineage a.d.5 was more frequently detected. These data are consistent with what was reported worldwide [54, 55] and supported by RSV phylogenetic trees with our sequences and all European sequences of those seasons (Figures S1–S4). Unfortunately, our sample size in the Sentinel ISN network is too small to find more correlations.

When comparing the RSV A circulating lineages between age‐matched groups across the different networks, we observed that most of the children < 3 months old were infected by the a.d.3 lineage, whereas in RSV B, B.D.E.1 lineage was predominant in all age groups. Due to the lower statistical power, we could not find more correlations.

From 2021 to 2023, a significant genetic variability was observed, with several lineages from both subgroups in cocirculation. These results show the real importance of the genetic characterization of RSV for national/global surveillance. In detail, Lineage a.d.5 was more frequent in 2021/2022, and regional clusters were detected in Lisboa e Vale do Tejo. In the subsequent season, regional clusters were detected in Lineage a.d.3 in the Norte and Lisboa e Vale do Tejo regions. The a.d.1 lineage was detected in all regions and both seasons. Regarding Subgroup B, several clusters were detected in Lineage B.D.E.1, indicating a high intralineage diversity level. All of these genetic clusters were also verified with Figures S1–S4, in which we challenged our sequences against all European sequences of respective seasons, and we could verify that all of our sequences clustered with the European ones, meaning that all strains detected in Portugal were detected in other countries.

It is worth noting that this study had some limitations. Although the surveillance systems continued to be active during the COVID‐19 pandemic, in the 2021/2022 season, there was a notably low number of RSV transmissions compared to the 2022/2023 season. Furthermore, the sample size obtained from both networks was not the same to make a good comparison, with 619 RSV‐positive samples received from the VigiRSV and 94 from Sentinel ISN, which was a very low sample size that ended up limiting our genomic correlations. Despite all regions presenting hospitals participating in the surveillance network, the number of cases reported was not representative of the population of each region. It is necessary to implement some encouragement measures to ensure equitable physician participation across regions and to make the samples collected more accurately represent hospitalized cases in the country and regions. Despite all of this, both systems showed a similar frequency of RSV subgroups, which could be a positive quality indicator of the RSV National Surveillance Systems. Given the high diversity of RSV within the country, this study also described the existence of regional clusters in Portugal during two seasons. Therefore, surveillance systems have the potential to monitor RSV activity and genomic evolution. Our study is based on the partial G gene, and because of that, our results should be considered with great caution and with a certain uncertainty. According to the RGCC [14] guidelines, using only part of this gene reduces the phylogenetic signal, which could result in a loss of classification precision. In addition to that, Nextclade is built for full genomes or full G gene sequences, and using it with partial input reduces the accuracy and confidence of these results. Despite this limitation, the results presented remain highly relevant and comparable to previous studies, especially considering the lack of genetic data since 2018 in Portugal and the current demand for information on RSV genetic diversity.

In summary, this study contributes to RSV knowledge on epidemiology and strain circulation in Portugal in the first post‐COVID‐19 Pandemic period (2021/2022 and 2022/2023 seasons). RSV showed a cocirculation of both subgroups during the study period, with a variability of nine lineages for Subgroup A and five for Subgroup B. The comparison between two surveillance networks targeting different surveillance settings demonstrated that there was a difference between lineages in RSV A but not in Subgroup B. Additionally, this study exhibited distinct regional clusters of RSV during the entire period. These results showed the importance of ongoing national and global surveillance of respiratory syncytial virus among other respiratory viruses, which will take on a major importance with the emergence of new lineages, especially with the arrival of new passive immunization methods and vaccines that will present new ways to protect young children.

## Author Contributions

**Miguel Lança:** writing – original draft, investigation, methodology, formal analysis, conceptualization, visualization. **Vânia Gaio:** writing – review and editing, formal analysis, visualization. **Ana Paula Rodrigues:** formal analysis, writing – review and editing, visualization. **Camila Henriques:** writing – review and editing, methodology, visualization. **Licínia Gomes:** methodology, writing – review and editing, visualization. **Daniela Dias:** methodology, writing – review and editing, visualization. **Maria de Jesus Chasqueira:** writing – review and editing, supervision, conceptualization, visualization. **Raquel Guiomar:** writing – review and editing, supervision, formal analysis, conceptualization, project administration, visualization. **Aryse Melo:** supervision, methodology, writing – review and editing, formal analysis, conceptualization, project administration, validation, visualization, investigation.

## Conflicts of Interest

The authors declare no conflicts of interest.

## Peer Review

The peer review history for this article is available at https://www.webofscience.com/api/gateway/wos/peer‐review/10.1111/irv.70147.

## Supporting information


**Table S1:** Reported Acute Respiratory Infection (ARI) cases, laboratory‐confirmed RSV cases and positivity rate detected in VigiRSV network, by region, age and gender.
**Table S2:** Reported Acute Respiratory Infection (ARI) cases and laboratory confirmed RSV cases detected in Sentinel ISN, by region, age and gender.
**Table S3:** Overview of the clinical presentations from all RSV‐positive samples received at LNRVG of INSA divided into the presence or absence of symptoms data through the National Respiratory Syncytial Virus Surveillance network (VigiRSV) and the Sentinel Influenza and other respiratory viruses Surveillance network (Sentinel ISN).
**Figure S1:** RSV A phylogenetic tree based on the C‐terminal second hypervariable region (HVR2) of the G gene, of 2021/2022 season with our Portuguese sequences and all European GISAID sequences in the same period. The phylogenetic tree was generated using the maximum likelihood method. The reliability of sequence clusters was evaluated with SH‐aLRT (1000 replicates) and UFBoot2 (1000 replicates). Only values of SH‐aLRT ≥ 80% and UFBoot2 ≥ 90% were represented at the branch nodes. Portuguese sequences are indicated in highlighted colour in the names. Reference sequences are indicated in black circles and are listed in the supplementary materials (Table S4). The phylogeny distribution can be visualized at https://itol.embl.de/export/19421015412593291750933220 using iTOL v6 (https://itol.embl.de/).
**Figure S2:** RSV A phylogenetic tree based on the C‐terminal second hypervariable region (HVR2) of the G gene, of 2022/2023 season with our Portuguese sequences and all European GISAID sequences in the same period. The phylogenetic tree was generated using the maximum likelihood method. The reliability of sequence clusters was evaluated with SH‐aLRT (1000 replicates) and UFBoot2 (1000 replicates). Only values of SH‐aLRT ≥ 80% and UFBoot2 ≥ 90% were represented at the branch nodes. Portuguese sequences are indicated in highlighted colour in the names. Reference sequences are indicated in black circles and are listed in the supplementary materials (Table S4). The phylogeny distribution can be visualized at https://itol.embl.de/export/1931379569191661751018860 using iTOL v6 (https://itol.embl.de/).
**Figure S3:** RSV B phylogenetic tree based on the C‐terminal second hypervariable region (HVR2) of the G gene, of 2021/2022 season with our Portuguese sequences and all European GISAID sequences in the same period. The phylogenetic tree was generated using the maximum likelihood method. The reliability of sequence clusters was evaluated with SH‐aLRT (1000 replicates) and UFBoot2 (1000 replicates). Only values of SH‐aLRT ≥ 80% and UFBoot2 ≥ 90% were represented at the branch nodes. Portuguese sequences are indicated in highlighted colour in the names. Reference sequences are indicated in black circles and are listed in the supplementary materials (Table S5). The phylogeny distribution can be visualized at https://itol.embl.de/export/19421015412594411750933242 using iTOL v6 (https://itol.embl.de/).
**Figure S4:** RSV B phylogenetic tree based on the C‐terminal second hypervariable region (HVR2) of the G gene, of 2022/2023 season with our Portuguese sequences and all European GISAID sequences in the same period. The phylogenetic tree was generated using the maximum likelihood method. The reliability of sequence clusters was evaluated with SH‐aLRT (1000 replicates) and UFBoot2 (1000 replicates). Only values of SH‐aLRT ≥ 80% and UFBoot2 ≥ 90% were represented at the branch nodes. Portuguese sequences are indicated in highlighted colour in the names. Reference sequences are indicated in black circles and are listed in the supplementary materials (Table S5). The phylogeny distribution can be visualized at https://itol.embl.de/export/1931379569191691751018860 using iTOL v6 (https://itol.embl.de/).
**Table S4:** List of accession numbers for the RSV A isolate sequences deposited in the NCBI GenBank database, with the corresponding lineage assigned to each genome.
**Table S5:** List of accession numbers for the RSV B isolate sequences deposited in the NCBI GenBank database, with the corresponding lineage assigned to each genome.
**Table S6:** GISAID accession numbers for all Portuguese sequences used in this study.

## Data Availability

The data that support the findings of this study are available from the corresponding author upon reasonable request.
