# Supplementary material for: Genetic and Epidemiological Insights Into Respiratory Syncytial Virus Infections: A Comparative Study of Hospitalized Versus Community Cases in Portugal (2021–2023)
Source: Influenza Other Respir Viruses. 2025 Oct 13;19(10):e70147. doi: 10.1111/irv.70147 (PMC12516350; doi:10.1111/irv.70147)
Supplement: Supplementary file 1 — Table S1: Reported Acute Respiratory Infection (ARI) cases, laboratory‐confirmed RSV cases and positivity rate detected in VigiRSV network, by region, age and gender. Table S2: Reported Acute Respiratory Infection (ARI) cases and laboratory confirmed RSV cases detected in Sentinel ISN, by region, age and gender. Table S3: Overview of the clinical presentations from all RSV‐positive samples received at LNRVG of INSA divided into the presence or absence of symptoms data through the National Respiratory Syncytial Virus Surveillance network (VigiRSV) and the Sentinel Influenza and other respiratory viruses Surveillance network (Sentinel ISN). Figure S1: RSV A phylogenetic tree based on the C‐terminal second hypervariable region (HVR2) of the G gene, of 2021/2022 season with our Portuguese sequences and all European GISAID sequences in the same period. The phylogenetic tree was generated using the maximum likelihood method. The reliability of sequence clusters was evaluated with SH‐aLRT (1000 replicates) and UFBoot2 (1000 replicates). Only values of SH‐aLRT ≥ 80% and UFBoot2 ≥ 90% were represented at the branch nodes. Portuguese sequences are indicated in highlighted colour in the names. Reference sequences are indicated in black circles and are listed in the supplementary materials (Table S4). The phylogeny distribution can be visualized at https://itol.embl.de/export/19421015412593291750933220 using iTOL v6 (https://itol.embl.de/). Figure S2: RSV A phylogenetic tree based on the C‐terminal second hypervariable region (HVR2) of the G gene, of 2022/2023 season with our Portuguese sequences and all European GISAID sequences in the same period. The phylogenetic tree was generated using the maximum likelihood method. The reliability of sequence clusters was evaluated with SH‐aLRT (1000 replicates) and UFBoot2 (1000 replicates). Only values of SH‐aLRT ≥ 80% and UFBoot2 ≥ 90% were represented at the branch nodes. Portuguese sequences are indicated in highlighted colour in the [file IRV-19-e70147-s001.docx]

**Supplementing Table 1.** Reported Acute Respiratory Infection (ARI) cases, laboratory-confirmed RSV cases and positivity rate detected in VigiRSV network, by region, age and gender.

|  | **VigiRSV network** | | | |
| --- | --- | --- | --- | --- |
|  | **Respiratory infection reported cases** | **Laboratory-confirmed RSV-positive cases** | **Positivity rateᵃ** | **RSV-positive samples received** |
| **Total** | **1569** | **892** | **56.9%** | **619** |
|  | **n (%)** | **n (%)** | **%** | **n (%)** |
| **Regions** |  |  |  |  |
| Alentejo | 141 (9.0%) | 85 (9.5%) | 60.3% | 51 (8.2%) |
| Algarve | 66 (4.2%) | 64 (7.2%) | 97.0% | 64 (10.3%) |
| Centro | 276 (17.6%) | 110 (12.3%) | 39.9% | 44 (7.1%) |
| Lisboa e Vale do Tejo | 518 (33.0%) | 392 (43.9%) | 75.7% | 280 (45.2%) |
| Norte | 568 (36.2%) | 241 (27.0%) | 42.4% | 181 (29.2%) |
| **Age group (months)** |  |  |  |  |
| < 3 | 727 (46.3%) | 459 (51.5%) | 63.1% | 327 (52.8%) |
| 3 - 5 | 263 (16.8%) | 169 (18.9%) | 64.3% | 122 (19.7%) |
| 6 - 11 | 292 (18.6%) | 155 (17.4%) | 53.1% | 100 (16.2%) |
| 12 - 23 | 287 (18.3%) | 109 (12.2%) | 38.0% | 70 (11.3%) |
| **Gender** |  |  |  |  |
| Male | 867 (55.3%) | 486 (54.5%) | 56.1% | 343 (55.4%) |
| Female | 702 (44.7%) | 406 (45.5%) | 57.8% | 276 (44.6%) |

ᵃPositivity rate was calculated between each subclass.

**Supplementing Table 2.** Reported Acute Respiratory Infection (ARI) cases and laboratory confirmed RSV cases detected in Sentinel ISN, by region, age and gender.

|  | **Sentinel ISN network** | | | |
| --- | --- | --- | --- | --- |
|  | **Respiratory infection reported cases** | **Laboratory-confirmed RSV-positive cases** | **Positivity rateᵃ** | **RSV-positive samples received** |
| **Total** | **1594** | **94** | **5.9%** | **94** |
|  | **n (%)** | **n (%)** | **%** | **n (%)** |
| **Regions** |  |  |  |  |
| Alentejo | 225 (14.1%) | 10 (10.6%) | 4.4% | 10 (10.6%) |
| Algarve | 30 (1.9%) | 3 (3.2%) | 10.0% | 3 (3.2%) |
| Centro | 178 (11.2%) | 16 (17.0%) | 9.0% | 16 (17.0%) |
| Lisboa e Vale do Tejo | 262 (16.4%) | 26 (27.7%) | 9.9% | 26 (27.7%) |
| Norte | 805 (50.5%) | 39 (41.5%) | 4.8% | 39 (41.5%) |
| **Age group (years)** |  |  |  |  |
| < 3 | 16 (1.0%) | 2 (2.1%) | 12.5% | 2 (2.1%) |
| 3 - 17 | 221 (13.9%) | 12 (12.8%) | 5.4% | 12 (12.8%) |
| 18 - 64 | 1135 (71.2%) | 54 (57.4%) | 4.8% | 54 (57.4%) |
| ≥ 65 | 222 (13.9%) | 26 (27.7%) | 11.7% | 26 (27.7%) |
| **Gender** |  |  |  |  |
| Male | 601 (37.7%) | 33 (35.1%) | 5.5% | 33 (35.1%) |
| Female | 993 (62.3%) | 61 (64.9%) | 6.1% | 61 (64.9%) |

ᵃPositivity rate was calculated between each subclass.

**Supplementing Table 3.** Overview of the clinical presentations from all RSV-positive samples received at LNRVG of INSA divided into the presence or absence of symptoms data through the National Respiratory Syncytial Virus Surveillance network (VigiRSV) and the Sentinel Influenza and other respiratory viruses Surveillance network (Sentinel ISN).

|  |  | **VigiRSV network** |  | **Sentinel ISN network** |
| --- | --- | --- | --- | --- |
|  | **N** | **n (%)** |  | **n (%)** |
| **Total** | 713 | **619** |  | **94** |
| **Clinical presentations** |  |  |  |  |
| **Cough** |  |  |  | |
| Data available |  | 48 (7.8%) |  | 88 (93.4%) |
| Unknown |  | 571 (92.2%) |  | 6 (6.4%) |
|  |  |  |  |  |
| **Sore throat** |  |  |  |  |
| Data available |  | 63 (10.2%) |  | 88 (93.4%) |
| Unknown |  | 556 (89.8%) |  | 6 (6.4%) |
|  |  |  |  |  |
| **Fever** |  |  |  |  |
| Data available |  | 48 (7.8%) |  | 89 (94.7%) |
| Unknown |  | 571 (92.2%) |  | 5 (5.3%) |
|  |  |  |  |  |
| **Shortness of breath** |  |  |  |  |
| Data available |  | 48 (7.8%) |  | 88 (93.4%) |
| Unknown |  | 571 (92.2%) |  | 6 (6.4%) |


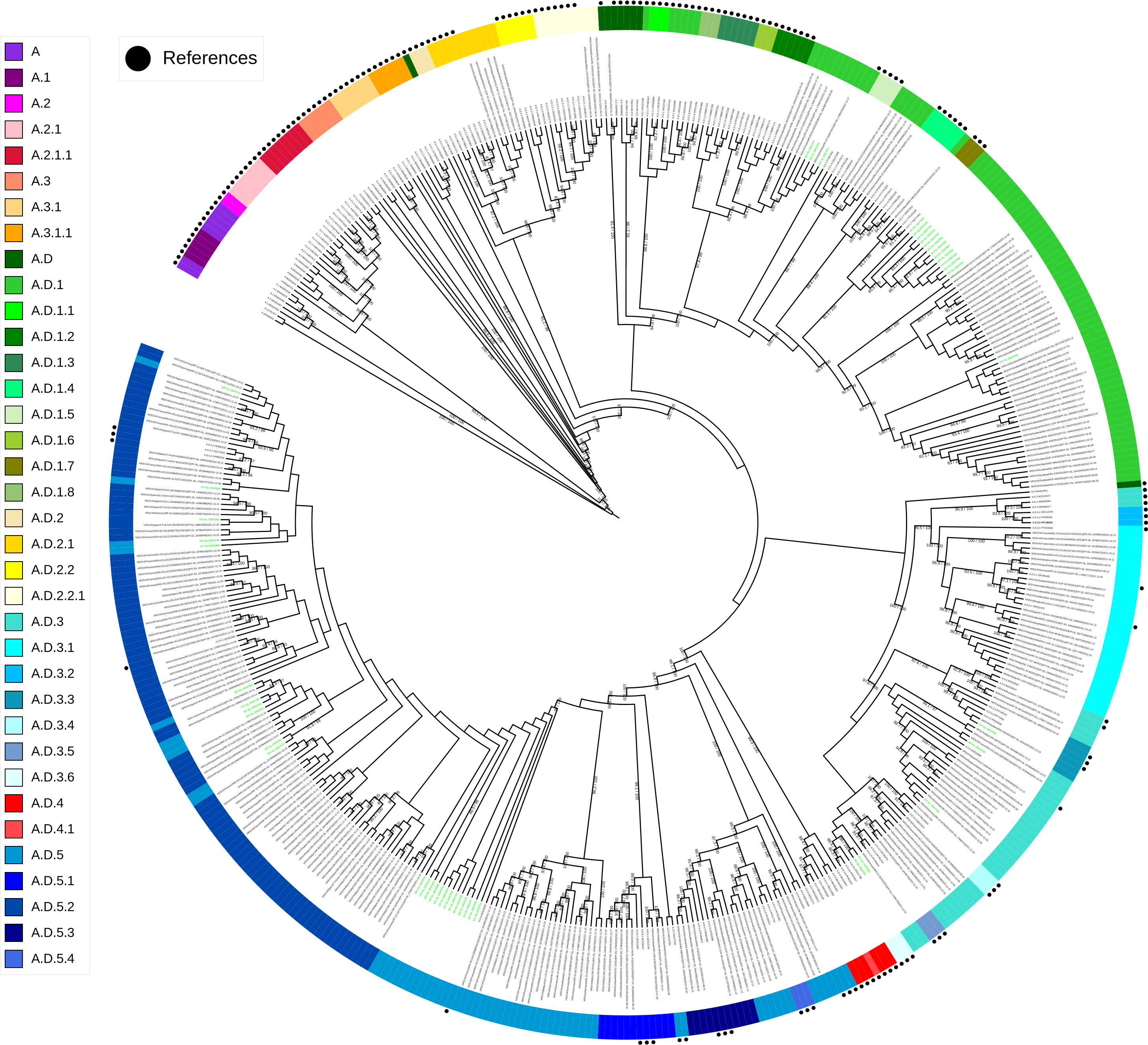


**Supplementing Figure 1.** RSV A phylogenetic tree based on the C-terminal second hypervariable region (HVR2) of the G gene, of 2021/2022 season with our Portuguese sequences and all European GISAID sequences in the same period. The phylogenetic tree was generated using the maximum likelihood method. The reliability of sequence clusters was evaluated with SH-aLRT (1000 replicates) and UFBoot2 (1000 replicates). Only values of SH-aLRT ≥ 80% and UFBoot2 ≥ 90% were represented at the branch nodes. Portuguese sequences are indicated in highlighted colour in the names. Reference sequences are indicated in black circles and are listed in the supplementary materials (Table S4). The phylogeny distribution can be visualized at <https://itol.embl.de/export/19421015412593291750933220> using iTOL v6 (<https://itol.embl.de/>).


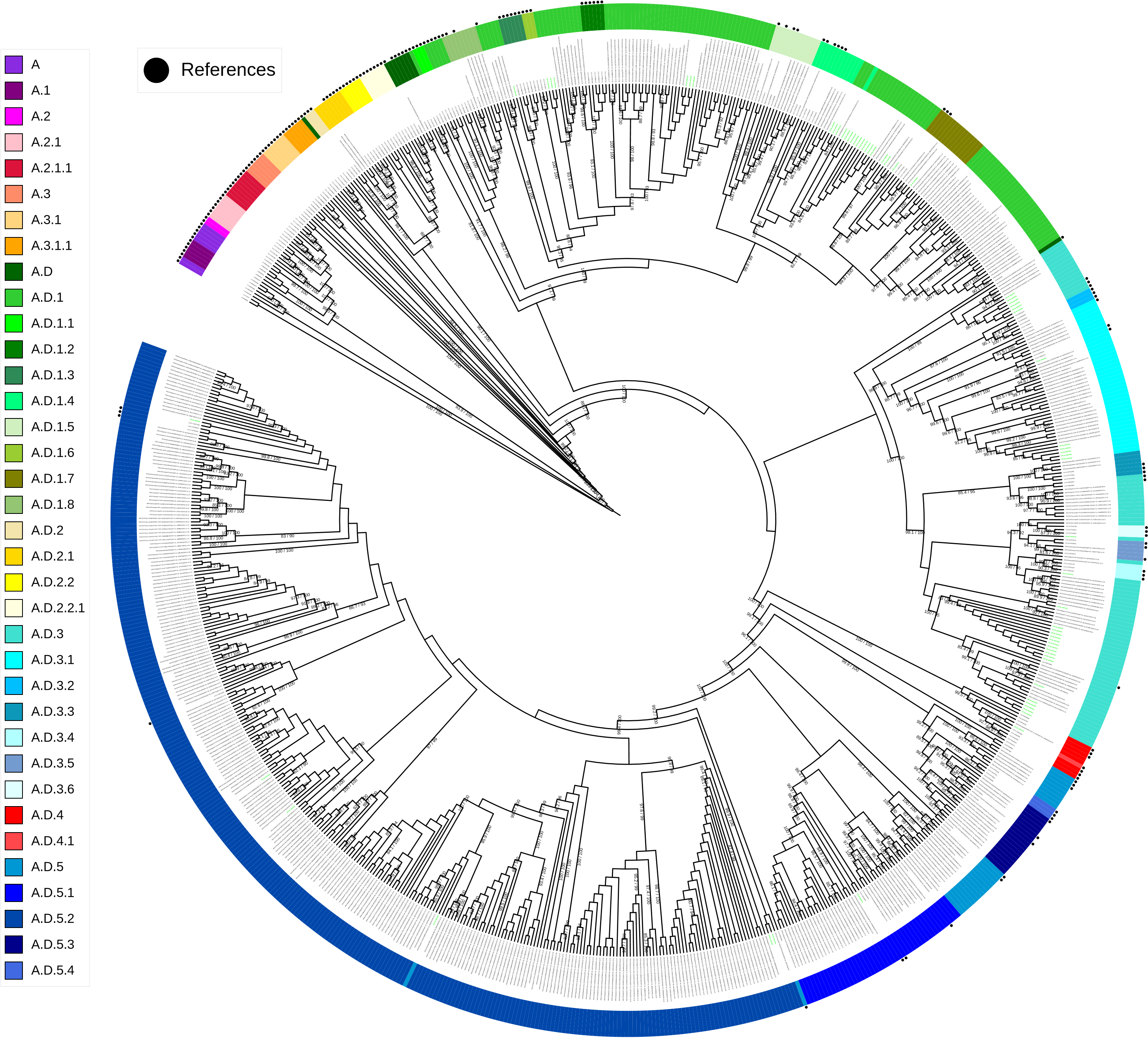


**Supplementing Figure 2.** RSV A phylogenetic tree based on the C-terminal second hypervariable region (HVR2) of the G gene, of 2022/2023 season with our Portuguese sequences and all European GISAID sequences in the same period. The phylogenetic tree was generated using the maximum likelihood method. The reliability of sequence clusters was evaluated with SH-aLRT (1000 replicates) and UFBoot2 (1000 replicates). Only values of SH-aLRT ≥ 80% and UFBoot2 ≥ 90% were represented at the branch nodes. Portuguese sequences are indicated in highlighted colour in the names. Reference sequences are indicated in black circles and are listed in the supplementary materials (Table S4). The phylogeny distribution can be visualized at <https://itol.embl.de/export/1931379569191661751018860> using iTOL v6 (<https://itol.embl.de/>).


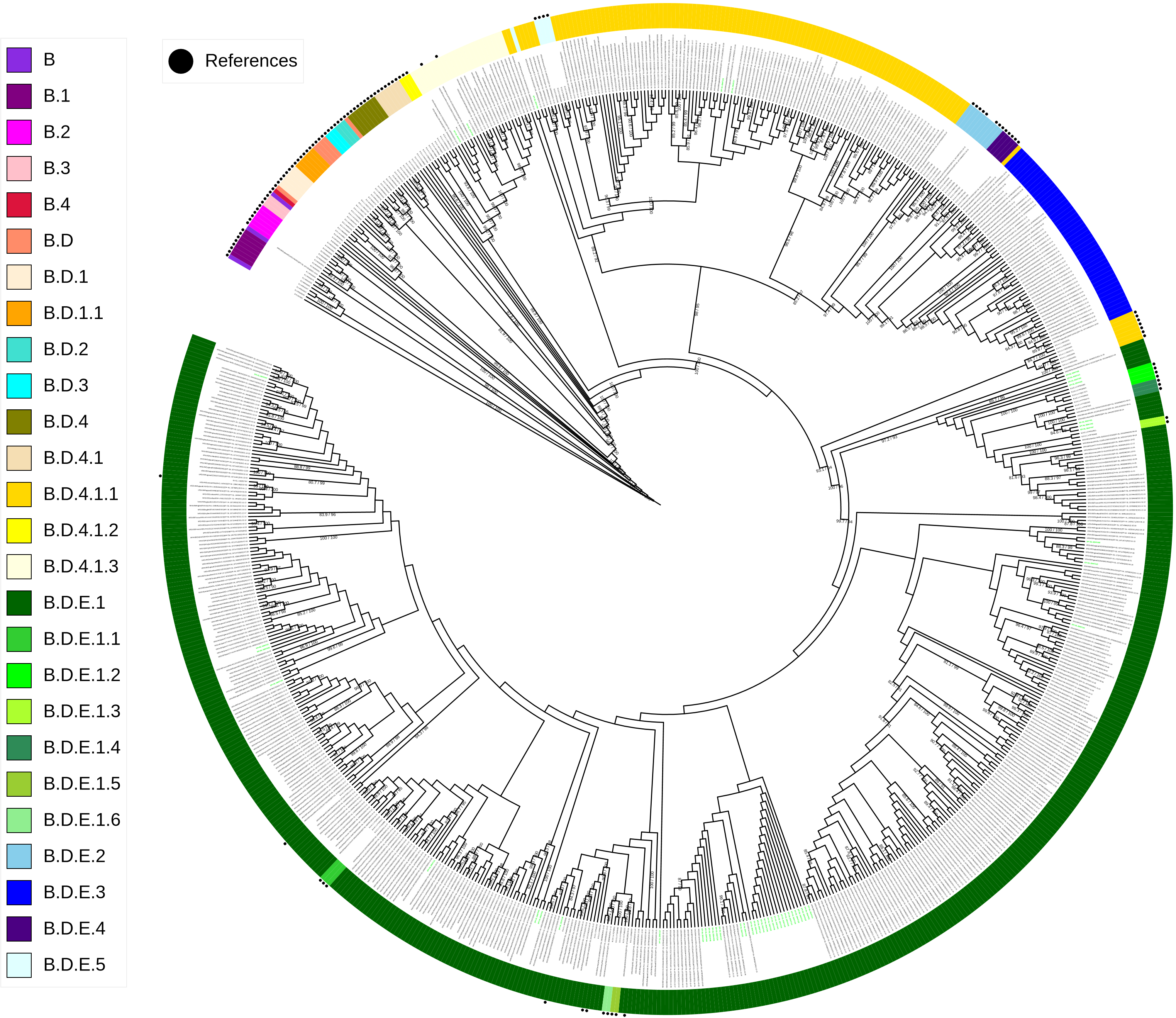


**Supplementing Figure 3.** RSV B phylogenetic tree based on the C-terminal second hypervariable region (HVR2) of the G gene, of 2021/2022 season with our Portuguese sequences and all European GISAID sequences in the same period. The phylogenetic tree was generated using the maximum likelihood method. The reliability of sequence clusters was evaluated with SH-aLRT (1000 replicates) and UFBoot2 (1000 replicates). Only values of SH-aLRT ≥ 80% and UFBoot2 ≥ 90% were represented at the branch nodes. Portuguese sequences are indicated in highlighted colour in the names. Reference sequences are indicated in black circles and are listed in the supplementary materials (Table S5). The phylogeny distribution can be visualized at <https://itol.embl.de/export/19421015412594411750933242> using iTOL v6 (<https://itol.embl.de/>).


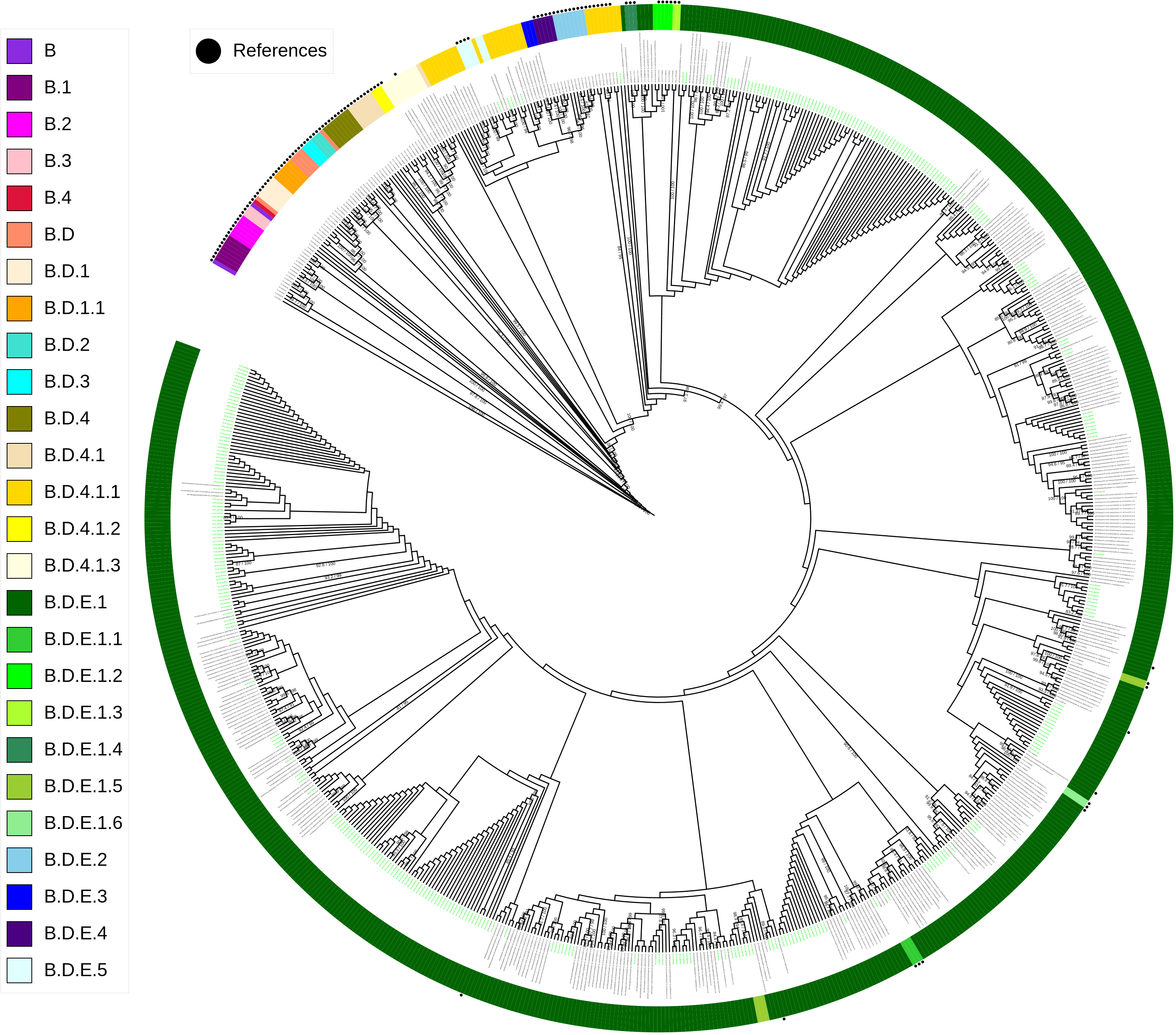


**Supplementing Figure 4.** RSV B phylogenetic tree based on the C-terminal second hypervariable region (HVR2) of the G gene, of 2022/2023 season with our Portuguese sequences and all European GISAID sequences in the same period. The phylogenetic tree was generated using the maximum likelihood method. The reliability of sequence clusters was evaluated with SH-aLRT (1000 replicates) and UFBoot2 (1000 replicates). Only values of SH-aLRT ≥ 80% and UFBoot2 ≥ 90% were represented at the branch nodes. Portuguese sequences are indicated in highlighted colour in the names. Reference sequences are indicated in black circles and are listed in the supplementary materials (Table S5). The phylogeny distribution can be visualized at <https://itol.embl.de/export/1931379569191691751018860> using iTOL v6 (<https://itol.embl.de/>).

**Supplementing Table 4.** List of accession numbers for the RSV A isolate sequences deposited in the NCBI GenBank database, with the corresponding lineage assigned to each genome.

| **RSV Subgroup** | **Lineage** | **NCBI ID**^1^ |  |
| --- | --- | --- | --- |
|  |  |  |  |
| A | A | MG642074 |  |
| A | A | MG642040 |  |
| A | A | MG642058 |  |
| A | A | MG642032 |  |
| A | A | MG642024 |  |
| A | A | LC741417 |  |
| A | A | OK649668 |  |
| A | A.1 | MG642060 |  |
| A | A.1 | MG642038 |  |
| A | A.1 | MG642069 |  |
| A | A.1 | MG642075 |  |
| A | A.1 | MG642067 |  |
| A | A.2 | MG642079 |  |
| A | A.2 | MG642083 |  |
| A | A.2.1 | KJ627676 |  |
| A | A.2.1 | KJ627708 |  |
| A | A.2.1 | KJ627672 |  |
| A | A.2.1 | KJ627652 |  |
| A | A.2.1 | KJ627670 |  |
| A | A.2.1 | KJ627671 |  |
| A | A.2.1 | KJ627663 |  |
| A | A.2.1.1 | KJ627717 |  |
| A | A.2.1.1 | KJ627649 |  |
| A | A.2.1.1 | KF973338 |  |
| A | A.2.1.1 | KF973339 |  |
| A | A.2.1.1 | KF973335 |  |
| A | A.2.1.1 | KJ627662 |  |
| A | A.2.1.1 | KJ627714 |  |
| A | A.2.1.1. | KJ627666 |  |
| A | A.3 | MG642063 |  |
| A | A.3 | MG642026 |  |
| A | A.3 | MG642081 |  |
| A | A.3 | MG642034 |  |
| A | A.3 | MG642035 |  |
| A | A.3 | MG642071 |  |
| A | A.3.1 | OK649667 |  |
| A | A.3.1 | OK649636 |  |
| A | A.3.1 | OK649634 |  |
| A | A.3.1 | OK649618 |  |
| A | A.3.1 | OK649628 |  |
| A | A.3.1 | OK649638 |  |
| A | A.3.1. | OK649641 |  |
| A | A.3.1.1 | OK649639 |  |
| A | A.3.1.1 | OK649647 |  |
| A | A.3.1.1 | OK649640 |  |
| A | A.3.1.1 | KY654507 |  |
| A | A.3.1.1 | KY654510 |  |
| A | A.3.1.1 | KY654508 |  |
| A | A.D | MK167035 |  |
| A | A.D | MH447951 |  |
| A | A.D | MH447953 |  |
| A | A.D | MH447954 |  |
| A | A.D | OK649682 |  |
| A | A.D | OK649681 |  |
| A | A.D | MF614946 |  |
| A | A.D | KJ627735 |  |
| A | A.D.1 | KY967363 |  |
| A | A.D.1 | MZ221198 |  |
| A | A.D.1 | MZ516058 |  |
| A | A.D.1 | MZ515789 |  |
| A | A.D.1 | ON237343 |  |
| A | A.D.1 | ON237349 |  |
| A | A.D.1 | MZ516008 |  |
| A | A.D.1.1 | PP495899 |  |
| A | A.D.1.1 | OR287843 |  |
| A | A.D.1.1 | PP495919 |  |
| A | A.D.1.2 | OM857171 |  |
| A | A.D.1.2 | OM857156 |  |
| A | A.D.1.2 | OM857174 |  |
| A | A.D.1.2 | OM857188 |  |
| A | A.D.1.2 | OM857177 |  |
| A | A.D.1.2 | OM857189 |  |
| A | A.D.1.3 | OM857301 |  |
| A | A.D.1.3 | OM857303 |  |
| A | A.D.1.3 | OM857334 |  |
| A | A.D.1.3 | OM857342 |  |
| A | A.D.1.3 | OM857338 |  |
| A | A.D.1.3 | OM857340 |  |
| A | A.D.1.4 | OQ024155 |  |
| A | A.D.1.4 | OR143137 |  |
| A | A.D.1.4 | PP681283 |  |
| A | A.D.1.4 | OY757595 |  |
| A | A.D.1.4 | PP352344 |  |
| A | A.D.1.4 | PP770472 |  |
| A | A.D.1.5 | OQ171906 |  |
| A | A.D.1.5 | OR143171 |  |
| A | A.D.1.5 | PP352349 |  |
| A | A.D.1.5 | OR883018 |  |
| A | A.D.1.6 | PP135017 |  |
| A | A.D.1.6 | PP795136 |  |
| A | A.D.1.6 | PP352345 |  |
| A | A.D.1.7 | OQ171911 |  |
| A | A.D.1.7 | PP203260 |  |
| A | A.D.1.7 | OR872616 |  |
| A | A.D.1.8 | PP973760 |  |
| A | A.D.1.8 | PP352324 |  |
| A | A.D.1.8 | OR287988 |  |
| A | A.D.2 | ON237272 |  |
| A | A.D.2 | MK749912 |  |
| A | A.D.2 | MK749913 |  |
| A | A.D.2.1 | MZ515901 |  |
| A | A.D.2.1 | MZ515859 |  |
| A | A.D.2.1 | MZ515701 |  |
| A | A.D.2.1 | MZ515709 |  |
| A | A.D.2.1 | MZ516080 |  |
| A | A.D.2.2 | ON237253 |  |
| A | A.D.2.2 | ON237273 |  |
| A | A.D.2.2 | MN630100 |  |
| A | A.D.2.2 | MK749890 |  |
| A | A.D.2.2 | MH383066 |  |
| A | A.D.2.2 | MH447958 |  |
| A | A.D.2.2.1 | ON237289 |  |
| A | A.D.2.2.1 | ON237294 |  |
| A | A.D.2.2.1 | ON237295 |  |
| A | A.D.2.2.1 | MZ515851 |  |
| A | A.D.2.2.1 | MZ515902 |  |
| A | A.D.2.2.1 | LR699737 |  |
| A | A.D.2.2.1 | MZ515575 |  |
| A | A.D.3 | MN306017 |  |
| A | A.D.3 | MN306045 |  |
| A | A.D.3 | MZ515619 |  |
| A | A.D.3 | MZ515647 |  |
| A | A.D.3 | MZ516063 |  |
| A | A.D.3 | MZ515668 |  |
| A | A.D.3.1 | PP376548 |  |
| A | A.D.3.1 | OR795445 |  |
| A | A.D.3.1 | OR522470 |  |
| A | A.D.3.2 | PP508181 |  |
| A | A.D.3.2 | OR143195 |  |
| A | A.D.3.2 | PP135024 |  |
| A | A.D.3.3 | PP748752 |  |
| A | A.D.3.3 | PP530269 |  |
| A | A.D.3.3 | PP770461 |  |
| A | A.D.3.4 | OQ261753 |  |
| A | A.D.3.4 | OQ024115 |  |
| A | A.D.3.4 | OR143204 |  |
| A | A.D.3.5 | OR795475 |  |
| A | A.D.3.5 | PP709453 |  |
| A | A.D.3.5 | PP781414 |  |
| A | A.D.3.6 | PP237786 |  |
| A | A.D.3.6 | PP401817 |  |
| A | A.D.3.6 | PP504647 |  |
| A | A.D.4 | MH181992 |  |
| A | A.D.4 | MH181991 |  |
| A | A.D.4 | MH182013 |  |
| A | A.D.4 | MH182020 |  |
| A | A.D.4 | MH182008 |  |
| A | A.D.4 | MH181997 |  |
| A | A.D.4.1 | MZ515682 |  |
| A | A.D.5 | ON237271 |  |
| A | A.D.5 | ON237320 |  |
| A | A.D.5 | MZ515825 |  |
| A | A.D.5 | MZ515782 |  |
| A | A.D.5 | MZ515592 |  |
| A | A.D.5 | MZ515884 |  |
| A | A.D.5.1 | MZ515567 |  |
| A | A.D.5.1 | MZ516012 |  |
| A | A.D.5.1 | MZ515749 |  |
| A | A.D.5.2 | OK500260 |  |
| A | A.D.5.2 | OP890339 |  |
| A | A.D.5.2 | OP890338 |  |
| A | A.D.5.2 | OQ171929 |  |
| A | A.D.5.3 | OP320399 |  |
| A | A.D.5.3 | PP495913 |  |
| A | A.D.5.3 | OR601480 |  |
| A | A.D.5.4 | PP135013 |  |
| A | A.D.5.4 | PP237781 |  |
| A | A.D.5.4 | PP781380 |  |

**Supplementing Table 5.** List of accession numbers for the RSV B isolate sequences deposited in the NCBI GenBank database, with the corresponding lineage assigned to each genome.

| **RSV Subgroup** | **Lineage** | **NCBI ID**^1^ |  |
| --- | --- | --- | --- |
|  |  |  |  |
| B | B | KU316097 |  |
| B | B | KP856965 |  |
| B | B.1 | MG642045 |  |
| B | B.1 | MG642037 |  |
| B | B.1 | MG642047 |  |
| B | B.1 | MG642065 |  |
| B | B.1 | MG642025 |  |
| B | B.1 | MG642044 |  |
| B | B.1 | MG642078 |  |
| B | B.2 | MG642043 |  |
| B | B.2 | MG642046 |  |
| B | B.2 | MG642057 |  |
| B | B.2 | MG642042 |  |
| B | B.2 | MG642039 |  |
| B | B.2 | MG642051 |  |
| B | B.3 | MG642049 |  |
| B | B.3 | MG642064 |  |
| B | B.3 | MG642062 |  |
| B | B.4 | KP258713 |  |
| B | B.D | OK649721 |  |
| B | B.D | OK649702 |  |
| B | B.D | OK649707 |  |
| B | B.D | MK109770 |  |
| B | B.D | OK649725 |  |
| B | B.D | MH594451 |  |
| B | B.D.1 | OK649699 |  |
| B | B.D.1 | MH594445 |  |
| B | B.D.1 | MH594450 |  |
| B | B.D.1 | MH594447 |  |
| B | B.D.1 | KY249659 |  |
| B | B.D.1 | KY249662 |  |
| B | B.D.1.1 | KY249674 |  |
| B | B.D.1.1 | KY249666 |  |
| B | B.D.1.1 | KY249661 |  |
| B | B.D.1.1 | ON237101 |  |
| B | B.D.1.1 | ON237110 |  |
| B | B.D.1.1 | ON237120 |  |
| B | B.D.2 | MH594443 |  |
| B | B.D.2 | MH594444 |  |
| B | B.D.2 | MH594461 |  |
| B | B.D.3 | MH594414 |  |
| B | B.D.3 | MH594418 |  |
| B | B.D.3 | MH594410 |  |
| B | B.D.4 | OK649704 |  |
| B | B.D.4 | OK649708 |  |
| B | B.D.4 | OK649728 |  |
| B | B.D.4 | OK649705 |  |
| B | B.D.4 | OK649726 |  |
| B | B.D.4 | KY249677 |  |
| B | B.D.4 | KY249667 |  |
| B | B.D.4 | KY249670 |  |
| B | B.D.4.1 | KY249660 |  |
| B | B.D.4.1 | LC384999 |  |
| B | B.D.4.1 | LC385005 |  |
| B | B.D.4.1 | ON237084 |  |
| B | B.D.4.1 | MK749871 |  |
| B | B.D.4.1 | MK749898 |  |
| B | B.D.4.1 | MK749892 |  |
| B | B.D.4.1.1 | ON237171 |  |
| B | B.D.4.1.1 | MZ515849 |  |
| B | B.D.4.1.1 | MZ515612 |  |
| B | B.D.4.1.1 | MZ515613 |  |
| B | B.D.4.1.1 | MZ515970 |  |
| B | B.D.4.1.1 | MZ515591 |  |
| B | B.D.4.1.1 | MZ515665 |  |
| B | B.D.4.1.2 | MN365320 |  |
| B | B.D.4.1.2 | MN365557 |  |
| B | B.D.4.1.2 | MN365519 |  |
| B | B.D.4.1.3 | OK500261 |  |
| B | B.D.4.1.3 | PQ762971 |  |
| B | B.D.E.1 | OP965700 |  |
| B | B.D.E.1 | OP965703 |  |
| B | B.D.E.1 | OP965707 |  |
| B | B.D.E.1 | OQ357797 |  |
| B | B.D.E.1 | OP890346 |  |
| B | B.D.E.1 | OP890343 |  |
| B | B.D.E.1.1 | PV081112 |  |
| B | B.D.E.1.1 | PQ492134 |  |
| B | B.D.E.1.1 | PV016988 |  |
| B | B.D.E.1.2 | PP910802 |  |
| B | B.D.E.1.2 | PP084063 |  |
| B | B.D.E.1.2 | PP959042 |  |
| B | B.D.E.1.2 | PQ117672 |  |
| B | B.D.E.1.3 | PP910821 |  |
| B | B.D.E.1.3 | PP709468 |  |
| B | B.D.E.1.4 | PP530270 |  |
| B | B.D.E.1.4 | PP957803 |  |
| B | B.D.E.1.4 | PV081093 |  |
| B | B.D.E.1.5 | PV081118 |  |
| B | B.D.E.1.5 | PV081119 |  |
| B | B.D.E.1.6 | PQ849400 |  |
| B | B.D.E.1.6 | PQ798811 |  |
| B | B.D.E.2 | MZ515558 |  |
| B | B.D.E.2 | MZ516135 |  |
| B | B.D.E.2 | MZ515938 |  |
| B | B.D.E.2 | MZ515997 |  |
| B | B.D.E.2 | MZ516102 |  |
| B | B.D.E.2 | OR666591 |  |
| B | B.D.E.2 | OM857390 |  |
| B | B.D.E.2 | OM857380 |  |
| B | B.D.E.4 | OM857389 |  |
| B | B.D.E.4 | OM857388 |  |
| B | B.D.E.4 | OM857382 |  |
| B | B.D.E.4 | OM857383 |  |
| B | B.D.E.4 | OM857387 |  |
| B | B.D.E.5 | OR975316 |  |
| B | B.D.E.5 | PQ638753 |  |
| B | B.D.E.5 | PQ083698 |  |
| B | B.D.E.5 | OR162288 |  |

**Supplementing Table 6.** GISAID accession numbers for all Portuguese sequences used in this study.

| **RSV Subgroup** | **Lineage** | **GISAID ID** |
| --- | --- | --- |
| A | A.D.1 | EPI_ISL_20047001 |
| A | A.D.1 | EPI_ISL_20047006 |
| A | A.D.1 | EPI_ISL_20047010 |
| A | A.D.1 | EPI_ISL_20047016 |
| A | A.D.1 | EPI_ISL_20047017 |
| A | A.D.1 | EPI_ISL_20047018 |
| A | A.D.1 | EPI_ISL_20047020 |
| A | A.D.1 | EPI_ISL_20047024 |
| A | A.D.1 | EPI_ISL_20047025 |
| A | A.D.1 | EPI_ISL_20047028 |
| A | A.D.1 | EPI_ISL_20047032 |
| A | A.D.1 | EPI_ISL_20047037 |
| A | A.D.1 | EPI_ISL_20047038 |
| A | A.D.1 | EPI_ISL_20047040 |
| A | A.D.1 | EPI_ISL_20047041 |
| A | A.D.1 | EPI_ISL_20047042 |
| A | A.D.1 | EPI_ISL_20047057 |
| A | A.D.1 | EPI_ISL_20047062 |
| A | A.D.1 | EPI_ISL_20047063 |
| A | A.D.1 | EPI_ISL_20047064 |
| A | A.D.1 | EPI_ISL_20047067 |
| A | A.D.1 | EPI_ISL_20047070 |
| A | A.D.1 | EPI_ISL_20047073 |
| A | A.D.1 | EPI_ISL_20047074 |
| A | A.D.1 | EPI_ISL_20047077 |
| A | A.D.1 | EPI_ISL_20047081 |
| A | A.D.1 | EPI_ISL_20047083 |
| A | A.D.1 | EPI_ISL_20047086 |
| A | A.D.1 | EPI_ISL_20047089 |
| A | A.D.1 | EPI_ISL_20047091 |
| A | A.D.1 | EPI_ISL_20047092 |
| A | A.D.1 | EPI_ISL_20047093 |
| A | A.D.1 | EPI_ISL_20047094 |
| A | A.D.1 | EPI_ISL_20047096 |
| A | A.D.1 | EPI_ISL_20047097 |
| A | A.D.1 | EPI_ISL_20047102 |
| A | A.D.1 | EPI_ISL_20047105 |
| A | A.D.1 | EPI_ISL_20047106 |
| A | A.D.1 | EPI_ISL_20047107 |
| A | A.D.1 | EPI_ISL_20047108 |
| A | A.D.1.7 | EPI_ISL_20047036 |
| A | A.D.3 | EPI_ISL_20047029 |
| A | A.D.3 | EPI_ISL_20047033 |
| A | A.D.3 | EPI_ISL_20047034 |
| A | A.D.3 | EPI_ISL_20047043 |
| A | A.D.3 | EPI_ISL_20047044 |
| A | A.D.3 | EPI_ISL_20047045 |
| A | A.D.3 | EPI_ISL_20047046 |
| A | A.D.3 | EPI_ISL_20047047 |
| A | A.D.3 | EPI_ISL_20047048 |
| A | A.D.3 | EPI_ISL_20047049 |
| A | A.D.3 | EPI_ISL_20047050 |
| A | A.D.3 | EPI_ISL_20047052 |
| A | A.D.3 | EPI_ISL_20047055 |
| A | A.D.3 | EPI_ISL_20047056 |
| A | A.D.3 | EPI_ISL_20047058 |
| A | A.D.3 | EPI_ISL_20047059 |
| A | A.D.3 | EPI_ISL_20047060 |
| A | A.D.3 | EPI_ISL_20047061 |
| A | A.D.3 | EPI_ISL_20047071 |
| A | A.D.3 | EPI_ISL_20047072 |
| A | A.D.3 | EPI_ISL_20047075 |
| A | A.D.3 | EPI_ISL_20047076 |
| A | A.D.3 | EPI_ISL_20047080 |
| A | A.D.3 | EPI_ISL_20047082 |
| A | A.D.3 | EPI_ISL_20047085 |
| A | A.D.3 | EPI_ISL_20047087 |
| A | A.D.3 | EPI_ISL_20047088 |
| A | A.D.3 | EPI_ISL_20047095 |
| A | A.D.3 | EPI_ISL_20047098 |
| A | A.D.3 | EPI_ISL_20047101 |
| A | A.D.3.1 | EPI_ISL_20047035 |
| A | A.D.3.1 | EPI_ISL_20047066 |
| A | A.D.3.1 | EPI_ISL_20047068 |
| A | A.D.3.1 | EPI_ISL_20047069 |
| A | A.D.3.1 | EPI_ISL_20047079 |
| A | A.D.3.1 | EPI_ISL_20047104 |
| A | A.D.3.3 | EPI_ISL_20047022 |
| A | A.D.4 | EPI_ISL_20047039 |
| A | A.D.5 | EPI_ISL_20046999 |
| A | A.D.5 | EPI_ISL_20047000 |
| A | A.D.5 | EPI_ISL_20047002 |
| A | A.D.5 | EPI_ISL_20047003 |
| A | A.D.5 | EPI_ISL_20047004 |
| A | A.D.5 | EPI_ISL_20047005 |
| A | A.D.5 | EPI_ISL_20047007 |
| A | A.D.5 | EPI_ISL_20047008 |
| A | A.D.5 | EPI_ISL_20047009 |
| A | A.D.5 | EPI_ISL_20047011 |
| A | A.D.5 | EPI_ISL_20047012 |
| A | A.D.5 | EPI_ISL_20047013 |
| A | A.D.5 | EPI_ISL_20047014 |
| A | A.D.5 | EPI_ISL_20047015 |
| A | A.D.5 | EPI_ISL_20047019 |
| A | A.D.5 | EPI_ISL_20047021 |
| A | A.D.5 | EPI_ISL_20047023 |
| A | A.D.5 | EPI_ISL_20047026 |
| A | A.D.5 | EPI_ISL_20047027 |
| A | A.D.5 | EPI_ISL_20047030 |
| A | A.D.5 | EPI_ISL_20047031 |
| A | A.D.5 | EPI_ISL_20047065 |
| A | A.D.5 | EPI_ISL_20047099 |
| A | A.D.5 | EPI_ISL_20047100 |
| A | A.D.5.1 | EPI_ISL_20047078 |
| A | A.D.5.2 | EPI_ISL_20047051 |
| A | A.D.5.2 | EPI_ISL_20047053 |
| A | A.D.5.2 | EPI_ISL_20047054 |
| A | A.D.5.2 | EPI_ISL_20047084 |
| A | A.D.5.2 | EPI_ISL_20047090 |
| A | A.D.5.2 | EPI_ISL_20047103 |
| B | B.D.4.1.1 | EPI_ISL_20047117 |
| B | B.D.4.1.1 | EPI_ISL_20047123 |
| B | B.D.4.1.1 | EPI_ISL_20047164 |
| B | B.D.4.1.1 | EPI_ISL_20047246 |
| B | B.D.4.1.1 | EPI_ISL_20047269 |
| B | B.D.4.1.1 | EPI_ISL_20047306 |
| B | B.D.4.1.1 | EPI_ISL_20047322 |
| B | B.D.4.1.3 | EPI_ISL_20047130 |
| B | B.D.4.1.3 | EPI_ISL_20047131 |
| B | B.D.E.1 | EPI_ISL_20047109 |
| B | B.D.E.1 | EPI_ISL_20047110 |
| B | B.D.E.1 | EPI_ISL_20047111 |
| B | B.D.E.1 | EPI_ISL_20047112 |
| B | B.D.E.1 | EPI_ISL_20047113 |
| B | B.D.E.1 | EPI_ISL_20047114 |
| B | B.D.E.1 | EPI_ISL_20047115 |
| B | B.D.E.1 | EPI_ISL_20047116 |
| B | B.D.E.1 | EPI_ISL_20047118 |
| B | B.D.E.1 | EPI_ISL_20047119 |
| B | B.D.E.1 | EPI_ISL_20047120 |
| B | B.D.E.1 | EPI_ISL_20047121 |
| B | B.D.E.1 | EPI_ISL_20047122 |
| B | B.D.E.1 | EPI_ISL_20047124 |
| B | B.D.E.1 | EPI_ISL_20047125 |
| B | B.D.E.1 | EPI_ISL_20047126 |
| B | B.D.E.1 | EPI_ISL_20047127 |
| B | B.D.E.1 | EPI_ISL_20047128 |
| B | B.D.E.1 | EPI_ISL_20047129 |
| B | B.D.E.1 | EPI_ISL_20047132 |
| B | B.D.E.1 | EPI_ISL_20047133 |
| B | B.D.E.1 | EPI_ISL_20047134 |
| B | B.D.E.1 | EPI_ISL_20047135 |
| B | B.D.E.1 | EPI_ISL_20047136 |
| B | B.D.E.1 | EPI_ISL_20047137 |
| B | B.D.E.1 | EPI_ISL_20047138 |
| B | B.D.E.1 | EPI_ISL_20047139 |
| B | B.D.E.1 | EPI_ISL_20047140 |
| B | B.D.E.1 | EPI_ISL_20047141 |
| B | B.D.E.1 | EPI_ISL_20047142 |
| B | B.D.E.1 | EPI_ISL_20047143 |
| B | B.D.E.1 | EPI_ISL_20047144 |
| B | B.D.E.1 | EPI_ISL_20047145 |
| B | B.D.E.1 | EPI_ISL_20047146 |
| B | B.D.E.1 | EPI_ISL_20047147 |
| B | B.D.E.1 | EPI_ISL_20047148 |
| B | B.D.E.1 | EPI_ISL_20047149 |
| B | B.D.E.1 | EPI_ISL_20047150 |
| B | B.D.E.1 | EPI_ISL_20047151 |
| B | B.D.E.1 | EPI_ISL_20047152 |
| B | B.D.E.1 | EPI_ISL_20047153 |
| B | B.D.E.1 | EPI_ISL_20047154 |
| B | B.D.E.1 | EPI_ISL_20047155 |
| B | B.D.E.1 | EPI_ISL_20047156 |
| B | B.D.E.1 | EPI_ISL_20047157 |
| B | B.D.E.1 | EPI_ISL_20047158 |
| B | B.D.E.1 | EPI_ISL_20047159 |
| B | B.D.E.1 | EPI_ISL_20047160 |
| B | B.D.E.1 | EPI_ISL_20047161 |
| B | B.D.E.1 | EPI_ISL_20047162 |
| B | B.D.E.1 | EPI_ISL_20047163 |
| B | B.D.E.1 | EPI_ISL_20047165 |
| B | B.D.E.1 | EPI_ISL_20047166 |
| B | B.D.E.1 | EPI_ISL_20047167 |
| B | B.D.E.1 | EPI_ISL_20047168 |
| B | B.D.E.1 | EPI_ISL_20047169 |
| B | B.D.E.1 | EPI_ISL_20047170 |
| B | B.D.E.1 | EPI_ISL_20047171 |
| B | B.D.E.1 | EPI_ISL_20047172 |
| B | B.D.E.1 | EPI_ISL_20047173 |
| B | B.D.E.1 | EPI_ISL_20047174 |
| B | B.D.E.1 | EPI_ISL_20047175 |
| B | B.D.E.1 | EPI_ISL_20047176 |
| B | B.D.E.1 | EPI_ISL_20047177 |
| B | B.D.E.1 | EPI_ISL_20047178 |
| B | B.D.E.1 | EPI_ISL_20047179 |
| B | B.D.E.1 | EPI_ISL_20047180 |
| B | B.D.E.1 | EPI_ISL_20047181 |
| B | B.D.E.1 | EPI_ISL_20047182 |
| B | B.D.E.1 | EPI_ISL_20047183 |
| B | B.D.E.1 | EPI_ISL_20047184 |
| B | B.D.E.1 | EPI_ISL_20047185 |
| B | B.D.E.1 | EPI_ISL_20047186 |
| B | B.D.E.1 | EPI_ISL_20047187 |
| B | B.D.E.1 | EPI_ISL_20047188 |
| B | B.D.E.1 | EPI_ISL_20047189 |
| B | B.D.E.1 | EPI_ISL_20047190 |
| B | B.D.E.1 | EPI_ISL_20047191 |
| B | B.D.E.1 | EPI_ISL_20047192 |
| B | B.D.E.1 | EPI_ISL_20047193 |
| B | B.D.E.1 | EPI_ISL_20047194 |
| B | B.D.E.1 | EPI_ISL_20047195 |
| B | B.D.E.1 | EPI_ISL_20047196 |
| B | B.D.E.1 | EPI_ISL_20047197 |
| B | B.D.E.1 | EPI_ISL_20047198 |
| B | B.D.E.1 | EPI_ISL_20047199 |
| B | B.D.E.1 | EPI_ISL_20047200 |
| B | B.D.E.1 | EPI_ISL_20047201 |
| B | B.D.E.1 | EPI_ISL_20047202 |
| B | B.D.E.1 | EPI_ISL_20047203 |
| B | B.D.E.1 | EPI_ISL_20047204 |
| B | B.D.E.1 | EPI_ISL_20047205 |
| B | B.D.E.1 | EPI_ISL_20047206 |
| B | B.D.E.1 | EPI_ISL_20047207 |
| B | B.D.E.1 | EPI_ISL_20047208 |
| B | B.D.E.1 | EPI_ISL_20047209 |
| B | B.D.E.1 | EPI_ISL_20047210 |
| B | B.D.E.1 | EPI_ISL_20047211 |
| B | B.D.E.1 | EPI_ISL_20047212 |
| B | B.D.E.1 | EPI_ISL_20047213 |
| B | B.D.E.1 | EPI_ISL_20047214 |
| B | B.D.E.1 | EPI_ISL_20047215 |
| B | B.D.E.1 | EPI_ISL_20047216 |
| B | B.D.E.1 | EPI_ISL_20047217 |
| B | B.D.E.1 | EPI_ISL_20047218 |
| B | B.D.E.1 | EPI_ISL_20047219 |
| B | B.D.E.1 | EPI_ISL_20047220 |
| B | B.D.E.1 | EPI_ISL_20047221 |
| B | B.D.E.1 | EPI_ISL_20047222 |
| B | B.D.E.1 | EPI_ISL_20047223 |
| B | B.D.E.1 | EPI_ISL_20047224 |
| B | B.D.E.1 | EPI_ISL_20047225 |
| B | B.D.E.1 | EPI_ISL_20047227 |
| B | B.D.E.1 | EPI_ISL_20047228 |
| B | B.D.E.1 | EPI_ISL_20047229 |
| B | B.D.E.1 | EPI_ISL_20047230 |
| B | B.D.E.1 | EPI_ISL_20047231 |
| B | B.D.E.1 | EPI_ISL_20047232 |
| B | B.D.E.1 | EPI_ISL_20047233 |
| B | B.D.E.1 | EPI_ISL_20047234 |
| B | B.D.E.1 | EPI_ISL_20047235 |
| B | B.D.E.1 | EPI_ISL_20047236 |
| B | B.D.E.1 | EPI_ISL_20047237 |
| B | B.D.E.1 | EPI_ISL_20047238 |
| B | B.D.E.1 | EPI_ISL_20047239 |
| B | B.D.E.1 | EPI_ISL_20047240 |
| B | B.D.E.1 | EPI_ISL_20047241 |
| B | B.D.E.1 | EPI_ISL_20047242 |
| B | B.D.E.1 | EPI_ISL_20047243 |
| B | B.D.E.1 | EPI_ISL_20047244 |
| B | B.D.E.1 | EPI_ISL_20047245 |
| B | B.D.E.1 | EPI_ISL_20047247 |
| B | B.D.E.1 | EPI_ISL_20047248 |
| B | B.D.E.1 | EPI_ISL_20047249 |
| B | B.D.E.1 | EPI_ISL_20047250 |
| B | B.D.E.1 | EPI_ISL_20047251 |
| B | B.D.E.1 | EPI_ISL_20047252 |
| B | B.D.E.1 | EPI_ISL_20047253 |
| B | B.D.E.1 | EPI_ISL_20047254 |
| B | B.D.E.1 | EPI_ISL_20047255 |
| B | B.D.E.1 | EPI_ISL_20047256 |
| B | B.D.E.1 | EPI_ISL_20047257 |
| B | B.D.E.1 | EPI_ISL_20047258 |
| B | B.D.E.1 | EPI_ISL_20047259 |
| B | B.D.E.1 | EPI_ISL_20047260 |
| B | B.D.E.1 | EPI_ISL_20047261 |
| B | B.D.E.1 | EPI_ISL_20047262 |
| B | B.D.E.1 | EPI_ISL_20047263 |
| B | B.D.E.1 | EPI_ISL_20047264 |
| B | B.D.E.1 | EPI_ISL_20047265 |
| B | B.D.E.1 | EPI_ISL_20047266 |
| B | B.D.E.1 | EPI_ISL_20047267 |
| B | B.D.E.1 | EPI_ISL_20047268 |
| B | B.D.E.1 | EPI_ISL_20047270 |
| B | B.D.E.1 | EPI_ISL_20047271 |
| B | B.D.E.1 | EPI_ISL_20047272 |
| B | B.D.E.1 | EPI_ISL_20047273 |
| B | B.D.E.1 | EPI_ISL_20047274 |
| B | B.D.E.1 | EPI_ISL_20047275 |
| B | B.D.E.1 | EPI_ISL_20047276 |
| B | B.D.E.1 | EPI_ISL_20047277 |
| B | B.D.E.1 | EPI_ISL_20047278 |
| B | B.D.E.1 | EPI_ISL_20047279 |
| B | B.D.E.1 | EPI_ISL_20047280 |
| B | B.D.E.1 | EPI_ISL_20047281 |
| B | B.D.E.1 | EPI_ISL_20047282 |
| B | B.D.E.1 | EPI_ISL_20047283 |
| B | B.D.E.1 | EPI_ISL_20047284 |
| B | B.D.E.1 | EPI_ISL_20047285 |
| B | B.D.E.1 | EPI_ISL_20047286 |
| B | B.D.E.1 | EPI_ISL_20047287 |
| B | B.D.E.1 | EPI_ISL_20047288 |
| B | B.D.E.1 | EPI_ISL_20047289 |
| B | B.D.E.1 | EPI_ISL_20047290 |
| B | B.D.E.1 | EPI_ISL_20047291 |
| B | B.D.E.1 | EPI_ISL_20047292 |
| B | B.D.E.1 | EPI_ISL_20047293 |
| B | B.D.E.1 | EPI_ISL_20047294 |
| B | B.D.E.1 | EPI_ISL_20047295 |
| B | B.D.E.1 | EPI_ISL_20047296 |
| B | B.D.E.1 | EPI_ISL_20047297 |
| B | B.D.E.1 | EPI_ISL_20047298 |
| B | B.D.E.1 | EPI_ISL_20047299 |
| B | B.D.E.1 | EPI_ISL_20047300 |
| B | B.D.E.1 | EPI_ISL_20047301 |
| B | B.D.E.1 | EPI_ISL_20047302 |
| B | B.D.E.1 | EPI_ISL_20047303 |
| B | B.D.E.1 | EPI_ISL_20047304 |
| B | B.D.E.1 | EPI_ISL_20047305 |
| B | B.D.E.1 | EPI_ISL_20047307 |
| B | B.D.E.1 | EPI_ISL_20047308 |
| B | B.D.E.1 | EPI_ISL_20047309 |
| B | B.D.E.1 | EPI_ISL_20047310 |
| B | B.D.E.1 | EPI_ISL_20047311 |
| B | B.D.E.1 | EPI_ISL_20047312 |
| B | B.D.E.1 | EPI_ISL_20047313 |
| B | B.D.E.1 | EPI_ISL_20047314 |
| B | B.D.E.1 | EPI_ISL_20047315 |
| B | B.D.E.1 | EPI_ISL_20047316 |
| B | B.D.E.1 | EPI_ISL_20047317 |
| B | B.D.E.1 | EPI_ISL_20047318 |
| B | B.D.E.1 | EPI_ISL_20047319 |
| B | B.D.E.1 | EPI_ISL_20047320 |
| B | B.D.E.1 | EPI_ISL_20047321 |
| B | B.D.E.1 | EPI_ISL_20047323 |
| B | B.D.E.1 | EPI_ISL_20047324 |
| B | B.D.E.1 | EPI_ISL_20047325 |
| B | B.D.E.1 | EPI_ISL_20047326 |
| B | B.D.E.1 | EPI_ISL_20047327 |
| B | B.D.E.1 | EPI_ISL_20047328 |
| B | B.D.E.1 | EPI_ISL_20047329 |
| B | B.D.E.1 | EPI_ISL_20047330 |
| B | B.D.E.1 | EPI_ISL_20047331 |
| B | B.D.E.1 | EPI_ISL_20047332 |
| B | B.D.E.1 | EPI_ISL_20047333 |
| B | B.D.E.1 | EPI_ISL_20047334 |
| B | B.D.E.1 | EPI_ISL_20047335 |
| B | B.D.E.1 | EPI_ISL_20047336 |
| B | B.D.E.1 | EPI_ISL_20047337 |
| B | B.D.E.1 | EPI_ISL_20047338 |
| B | B.D.E.1 | EPI_ISL_20047339 |
| B | B.D.E.1 | EPI_ISL_20047340 |
| B | B.D.E.1 | EPI_ISL_20047341 |
| B | B.D.E.1 | EPI_ISL_20047342 |
| B | B.D.E.1 | EPI_ISL_20047343 |
| B | B.D.E.1 | EPI_ISL_20047344 |
| B | B.D.E.1 | EPI_ISL_20047345 |
| B | B.D.E.1 | EPI_ISL_20047346 |
| B | B.D.E.1 | EPI_ISL_20047347 |
| B | B.D.E.1 | EPI_ISL_20047348 |
| B | B.D.E.1 | EPI_ISL_20047349 |
| B | B.D.E.1 | EPI_ISL_20047350 |
| B | B.D.E.1 | EPI_ISL_20047351 |
| B | B.D.E.1 | EPI_ISL_20047352 |
| B | B.D.E.1 | EPI_ISL_20047353 |
| B | B.D.E.1 | EPI_ISL_20047354 |
| B | B.D.E.1 | EPI_ISL_20047355 |
| B | B.D.E.1 | EPI_ISL_20047356 |
| B | B.D.E.1 | EPI_ISL_20047357 |
| B | B.D.E.1 | EPI_ISL_20047358 |
| B | B.D.E.1 | EPI_ISL_20047359 |
| B | B.D.E.1 | EPI_ISL_20047360 |
| B | B.D.E.1 | EPI_ISL_20047361 |
| B | B.D.E.1 | EPI_ISL_20047362 |
| B | B.D.E.1 | EPI_ISL_20047363 |
| B | B.D.E.1 | EPI_ISL_20047364 |
| B | B.D.E.1 | EPI_ISL_20047365 |
| B | B.D.E.1 | EPI_ISL_20047366 |
| B | B.D.E.1 | EPI_ISL_20047367 |
| B | B.D.E.1 | EPI_ISL_20047368 |
| B | B.D.E.1 | EPI_ISL_20047369 |
| B | B.D.E.1 | EPI_ISL_20047370 |
| B | B.D.E.1 | EPI_ISL_20047371 |
| B | B.D.E.1 | EPI_ISL_20047372 |
| B | B.D.E.1 | EPI_ISL_20047373 |
| B | B.D.E.1 | EPI_ISL_20047374 |
| B | B.D.E.1 | EPI_ISL_20047375 |
| B | B.D.E.1 | EPI_ISL_20047376 |
| B | B.D.E.1 | EPI_ISL_20047377 |
| B | B.D.E.1 | EPI_ISL_20047378 |
| B | B.D.E.1 | EPI_ISL_20047379 |
| B | B.D.E.1 | EPI_ISL_20047380 |
| B | B.D.E.1 | EPI_ISL_20047381 |
| B | B.D.E.1 | EPI_ISL_20047382 |
| B | B.D.E.1 | EPI_ISL_20047383 |
| B | B.D.E.1 | EPI_ISL_20047384 |
| B | B.D.E.1 | EPI_ISL_20047386 |
| B | B.D.E.1 | EPI_ISL_20047387 |
| B | B.D.E.1 | EPI_ISL_20047388 |
| B | B.D.E.1 | EPI_ISL_20047389 |
| B | B.D.E.1 | EPI_ISL_20047390 |
| B | B.D.E.1 | EPI_ISL_20047391 |
| B | B.D.E.1 | EPI_ISL_20047392 |
| B | B.D.E.1 | EPI_ISL_20047393 |
| B | B.D.E.1 | EPI_ISL_20047394 |
| B | B.D.E.1 | EPI_ISL_20047395 |
| B | B.D.E.1 | EPI_ISL_20047396 |
| B | B.D.E.1 | EPI_ISL_20047397 |
| B | B.D.E.1 | EPI_ISL_20047398 |
| B | B.D.E.1 | EPI_ISL_20047399 |
| B | B.D.E.1 | EPI_ISL_20047400 |
| B | B.D.E.1 | EPI_ISL_20047401 |
| B | B.D.E.1 | EPI_ISL_20047402 |
| B | B.D.E.1 | EPI_ISL_20047403 |
| B | B.D.E.1 | EPI_ISL_20047404 |
| B | B.D.E.1 | EPI_ISL_20047405 |
| B | B.D.E.1 | EPI_ISL_20047406 |
| B | B.D.E.1 | EPI_ISL_20047407 |
| B | B.D.E.1 | EPI_ISL_20047408 |
| B | B.D.E.1 | EPI_ISL_20047409 |
| B | B.D.E.1 | EPI_ISL_20047410 |
| B | B.D.E.1 | EPI_ISL_20047411 |
| B | B.D.E.1 | EPI_ISL_20047412 |
| B | B.D.E.1 | EPI_ISL_20047413 |
| B | B.D.E.1 | EPI_ISL_20047414 |
| B | B.D.E.1 | EPI_ISL_20047415 |
| B | B.D.E.1 | EPI_ISL_20047416 |
| B | B.D.E.1 | EPI_ISL_20047417 |
| B | B.D.E.1 | EPI_ISL_20047418 |
| B | B.D.E.1 | EPI_ISL_20047419 |
| B | B.D.E.1 | EPI_ISL_20047420 |
| B | B.D.E.1 | EPI_ISL_20047421 |
| B | B.D.E.1 | EPI_ISL_20047422 |
| B | B.D.E.1 | EPI_ISL_20047423 |
| B | B.D.E.1 | EPI_ISL_20047424 |
| B | B.D.E.1 | EPI_ISL_20047425 |
| B | B.D.E.1 | EPI_ISL_20047426 |
| B | B.D.E.1 | EPI_ISL_20047427 |
| B | B.D.E.1 | EPI_ISL_20047428 |
| B | B.D.E.1 | EPI_ISL_20047429 |
| B | B.D.E.1 | EPI_ISL_20047430 |
| B | B.D.E.1 | EPI_ISL_20047431 |
| B | B.D.E.1 | EPI_ISL_20047432 |
| B | B.D.E.1 | EPI_ISL_20047433 |
| B | B.D.E.1 | EPI_ISL_20047434 |
| B | B.D.E.1 | EPI_ISL_20047435 |
| B | B.D.E.1 | EPI_ISL_20047436 |
| B | B.D.E.1 | EPI_ISL_20047437 |
| B | B.D.E.1 | EPI_ISL_20047438 |
| B | B.D.E.1 | EPI_ISL_20047439 |
| B | B.D.E.1 | EPI_ISL_20047440 |
| B | B.D.E.1 | EPI_ISL_20047441 |
| B | B.D.E.1 | EPI_ISL_20047442 |
| B | B.D.E.1 | EPI_ISL_20047443 |
| B | B.D.E.1 | EPI_ISL_20047444 |
| B | B.D.E.1 | EPI_ISL_20047445 |
| B | B.D.E.1 | EPI_ISL_20047446 |
| B | B.D.E.1 | EPI_ISL_20047447 |
| B | B.D.E.1 | EPI_ISL_20047448 |
| B | B.D.E.1 | EPI_ISL_20047449 |
| B | B.D.E.1 | EPI_ISL_20047451 |
| B | B.D.E.1 | EPI_ISL_20047452 |
| B | B.D.E.1 | EPI_ISL_20047453 |
| B | B.D.E.1 | EPI_ISL_20047454 |
| B | B.D.E.1 | EPI_ISL_20047455 |
| B | B.D.E.1 | EPI_ISL_20047456 |
| B | B.D.E.1 | EPI_ISL_20047457 |
| B | B.D.E.1 | EPI_ISL_20047458 |
| B | B.D.E.1 | EPI_ISL_20047459 |
| B | B.D.E.1 | EPI_ISL_20047460 |
| B | B.D.E.1 | EPI_ISL_20047461 |
| B | B.D.E.1 | EPI_ISL_20047462 |
| B | B.D.E.1 | EPI_ISL_20047463 |
| B | B.D.E.1 | EPI_ISL_20047464 |
| B | B.D.E.1 | EPI_ISL_20047465 |
| B | B.D.E.1 | EPI_ISL_20047466 |
| B | B.D.E.1 | EPI_ISL_20047467 |
| B | B.D.E.1 | EPI_ISL_20047468 |
| B | B.D.E.1 | EPI_ISL_20047469 |
| B | B.D.E.1 | EPI_ISL_20047470 |
| B | B.D.E.1 | EPI_ISL_20047471 |
| B | B.D.E.1 | EPI_ISL_20047472 |
| B | B.D.E.1 | EPI_ISL_20047473 |
| B | B.D.E.1 | EPI_ISL_20047474 |
| B | B.D.E.1 | EPI_ISL_20047475 |
| B | B.D.E.1 | EPI_ISL_20047476 |
| B | B.D.E.1 | EPI_ISL_20047477 |
| B | B.D.E.1 | EPI_ISL_20047479 |
| B | B.D.E.1 | EPI_ISL_20047480 |
| B | B.D.E.1 | EPI_ISL_20047481 |
| B | B.D.E.1 | EPI_ISL_20047482 |
| B | B.D.E.1 | EPI_ISL_20047483 |
| B | B.D.E.1 | EPI_ISL_20047484 |
| B | B.D.E.1 | EPI_ISL_20047485 |
| B | B.D.E.1 | EPI_ISL_20047486 |
| B | B.D.E.1 | EPI_ISL_20047487 |
| B | B.D.E.1 | EPI_ISL_20047488 |
| B | B.D.E.1 | EPI_ISL_20047489 |
| B | B.D.E.1 | EPI_ISL_20047490 |
| B | B.D.E.1 | EPI_ISL_20047491 |
| B | B.D.E.1 | EPI_ISL_20047492 |
| B | B.D.E.1 | EPI_ISL_20047493 |
| B | B.D.E.1.5 | EPI_ISL_20047226 |
| B | B.D.E.1.5 | EPI_ISL_20047385 |
| B | B.D.E.1.5 | EPI_ISL_20047478 |
| B | B.D.E.5 | EPI_ISL_20047450 |

**Reference**

1. Neher, R., Goya, S. & Hinrichs, A. S. RSV Genotyping Consensus Consortium GitHub page. https://github.com/rsv-lineages/Classification_proposal.
